# Epigenetic Regulation of Cellular Senescence and Aging

**DOI:** 10.3389/fgene.2017.00138

**Published:** 2017-09-26

**Authors:** Corinne Sidler, Olga Kovalchuk, Igor Kovalchuk

**Affiliations:** Department of Biological Sciences, University of Lethbridge, Lethbridge, AB, Canada

**Keywords:** aging, cell senescence, epigenetic regulation, DNA methylation, heterochromatin, histone modifications

## Abstract

Aging is characterized by functional decline of diverse organs and an increased risk for several diseases. Therefore, a high interest exists in understanding the molecular mechanisms that stimulate aging at all levels, from cells and tissues to organs and organisms, in order to develop ways to promote healthy aging. While many molecular and biochemical mechanisms are already understood in some detail, the role of changes in epigenetic regulation has only begun to be considered in recent years. The age-dependent global reduction in heterochromatin, along with site-specific changes in the patterns of DNA methylation and modification of histones, have been observed in several aging model systems. However, understanding of the precise role of such changes requires further research. In this review, we will discuss the role of epigenetic regulation in aging and indicate future research directions that will help elucidate the mechanistic details of it.

## Introduction

Aging is an associated with an increased risk of several morbidities such cancer, cardiovascular disease, and autoimmune disease. It is associated with the body's altered capacity to cope with stress induced by metabolism, infection, and damage to cellular macromolecules. Understanding the molecular mechanisms that advance aging will help scientists to understand why aging individuals are more prone to those diseases and why they may be less stress-resistant.

The cells in the aging body exhibit large differences to cells in the juvenile organism, ranging from changed gene expression and DNA methylation patterns (Heyn et al., 2012) to shortened telomeres (Allsopp et al., 1992) and deteriorating genome maintenance mechanisms (Gorbunova et al., 2007). At the tissue, organ, and systemic levels, cells are exposed to altered stromal milieus caused by altered secretory profiles of senescent cells (Rodier et al., 2009) and a deteriorating immune system that is not able to mount immune responses toward new antigens equal to that of a younger immune system (MacKall et al., 1996). The combination of those factors may facilitate a malignant transformation of cells and prevent the efficient recognition and clearance of transformed cells; thus, the cancer incidence is increased.

In recent years, epigenetic mechanisms have been increasingly recognized for playing important roles in health and disease. In addition, age-dependent changes in epigenetic regulation have been observed indicating that epigenetic regulation may also play an important role in aging. The following paragraphs will examine the role of epigenetic regulation in aging and conclude by highlighting some of the open questions in the field.

## Aging: models and molecular mechanisms

Aging is characterized by the functional decline of the organism and an increase in a chance of dying at any time. In order to determine molecular mechanisms that underlie aging, the field of aging research has pursued two major directions: the study of lifespan modifications, which allows us to determine effects of gene mutations on median and maximum lifespan of an organism, and the study of functional senescence, which investigates the age-related intrinsic functional decline in the functional status of cells, organs, and organ systems (Grotewiel et al., 2005). Both types of approaches have identified several mechanisms that affect aging, including telomere shortening, nutrient sensing and signaling (caloric restriction, and mutations in related signaling pathways, such as IGF1 and insulin signaling, TOR signaling that leads to increased lifespans in several model organisms (Table S1), mitochondrial dysfunction and oxidative stress (which by causing damage to macromolecules of the cell may promote aging; on the other hand, mild mitochondrial dysfunction has also been linked to lifespan extension in lower organisms through an adaptive response termed mitohormesis), deterioration of DNA repair and accumulation of DNA damage, changes in protein homeostasis leading to the accumulation and aggregation of misfolded proteins, and changes in epigenetic regulation, which will be the topic of this review (Tables S1, S2 and references within).

The exact mechanism that causes organismal aging is not yet understood. A model has been proposed suggesting that the process of aging is not a clonal phenomenon, but rather the result of increased cell heterogeneity in a tissue (Vijg and Dolle, 2002). Thus, it is possible that the functional decline of tissue is caused by various changes to the cells it is composed of and, as a result, to the functional decline of the organism. Given the high specialization of all the organs that make up an organism, it is likely that the mechanisms that contribute to the functional decline of certain tissue or cell types also differ. Cells of the intestinal epithelium, which are constantly exposed to stress, are likely more susceptible to senescence caused by activated DNA damage signaling (Wang et al., 2009), whereas T-cells that need to clonally expand in order to perform their immune function are likely more prone to senescence due to telomere shortening (Bodnar et al., 1996).

Clearly, more research is needed to get a systemic understanding of the underlying mechanisms that cause aging. Understanding the processes that drive aging at a cell and organ level is a first step and has already given researchers insights into the commonalities and differences in the aging process of different organs. In order to potentially increase the functional lifespan of humans, it will be necessary to gain a systemic understanding of the aging process, including the identification of the initial molecular changes entailed in aging.

Below, we will summarize knowledge on the molecular mechanisms that underlie cellular senescence and promote aging at the cellular level. We will also examine mechanisms that underlie immunosenescence as an example for an aging system.

## Cellular senescence

Human diploid cells have a limited lifespan after which they terminally exit the cell cycle (Hayflick, 1965). This is termed cellular senescence. Cellular senescence is thought to play a role in aging, but this idea is still controversial. Several studies have shown that senescent cells accumulate in aging tissues *in vivo* (Herbig et al., 2006; Farr et al., 2016; Biran et al., 2017). Fibroblasts from progeria patients exhibit shortened replicative lifespans (Huang et al., 2008), suggesting that cellular senescence is linked with organismal aging. At the same time, the *in vitro* replicative lifespan of fibroblasts weakly correlates with donor age and the lifespan of the donor species (Smith et al., 2002; Ng et al., 2009). Assessment of 669 fibroblast cell cultures deriving from donors with different ages showed significant correlation between donor age and cell proliferative potential in females but not in males (Smith et al., 2002). The results of another study showed that telomerase activity and *in vitro* fibroblast cell growth were independent of donor age (Ng et al., 2009). Similarly, a study by Cristofalo et al. (1998) did not find any correlation between replicative lifespan of skin fibroblasts and donor age (Cristofalo et al., 1998).

Senescence may play a different role in different cell types during aging. Senescence typically occurs in the differentiated cells in most organs but can also be initiated in the stem cells influencing the capacity of tissues to renew and repair (Krishnamurthy et al., 2006). It is still unclear, however, whether the decrease in the functionality of stem cells that is observed with aging is the result of aging of stem cells or rather the result of changes in the niche, an environment that surrounds the cells in the aging tissue (Rando, 2006). The former was recently support by the observation that adult quiescent muscle stem cells undergo irreversible senescence in muscle tissues of old mice (Sousa-Victor et al., 2014). Thus, while senescent cells accumulate in tissues of aging individuals, the involvement of senescence in aging seems to be dependent on the affected cell types and tissues.

While senescence may promote aging and age-related pathologies, altered expression of senescence-associated genes may be sufficient to trigger carcinogenesis of human cells (Hahn et al., 1999). Hence, understanding of molecular mechanisms that underlie senescence is helpful in two ways: it will allow scientists to attempt to prolong the healthy lifespan, and potentially to develop new therapy of cancer.

To date, several mechanisms that promote senescence have been identified, which mainly involve accumulation of damage to DNA and other macromolecules of the cell (Lundblad and Szostak, 1989; Harley et al., 1990; Sohal and Weindruch, 1996; Vaziri and Benchimol, 1996; Sedelnikova et al., 2004; Seluanov et al., 2004; Sfeir and de Lange, 2012).

Furthermore, chromatin structure plays a critical role in regulation of cell and organism life span (Sedivy et al., 2008; Feser and Tyler, 2011; O'Sullivan and Karlseder, 2012). Decrease in the expression of genes encoding histones and the decrease in the number of repressive heterochromatin marks, such as methylation of DNA, and methylation of histones, including H3K9me3, H3K27me3, and H4K20me3 marks, observed in senescent cells and older organisms may suggest that aging at least in part is associated with the reduction of heterochromatin, which will be discussed in the sections that follow.

## Immunosenescence

Immunosenescence is the age-dependent deterioration of the immune system; it is linked with higher morbidity and mortality in the elderly population (Ferguson et al., 1995), as the immune system protects animals against pathogens and eliminates abnormally proliferating cells. In order to avoid autoimmune responses, maturing T-cells are subject to positive selection (T-cell are activated by antigen survive) and negative selection (autoreactive T-cells are reduced in numbers) in the thymus. These selection processes are not very efficient but energy-consuming, typically resulting in the elimination of over 95% of newly generated T-cells (Surh and Sprent, 1994). Therefore, when peripheral lymphatic organs are supplied with sufficient population of T-cells, the thymus shrinks, decreasing in size by about ~3% a year until fifth decades when it starts shrinking by ~1% per year (Steinmann et al., 1985). This phenomenon, often referred to as thymic involution, occurs similarly in all species that have thymus. Thymic involution also involves changes in the thymic cellularity and architecture leading to a decrease of production of naïve T-cells per the size of the thymus (Cunningham et al., 2001).

This reduced thymic T-cell output is paralleled by repeated clonal selection and expansion of T-cells in the periphery of an organism, resulting in an increased specialization and a decreased proliferative potential of the peripheral T-cell population. Both of these changes contribute to immunosenescence (Ferguson et al., 1995). Thus, immunosenescence differs from cellular senescence in that it involves the deterioration of a system rather than simply the terminal growth arrest of a single cell. However, cellular senescence may contribute mechanistically to immunosenescence, including thymic involution as outlined in the following paragraph.

The epithelial compartment of the thymus itself seems to play a crucial role in promoting thymic involution (Ortman et al., 2002; Zhu et al., 2007; Aw et al., 2009). During thymic involution, the epithelial compartment accumulates senescent and apoptotic cells (Aw et al., 2008) resulting in a shrinkage of the epithelial compartment (Flores et al., 1999). Consequently, cellular senescence and cell death may limit the functionality and the regeneration of the aging thymus and promote its age-dependent functional decline. On the other hand, the peripheral T-cell pool that is subject to repeated clonal expansions is prone to telomere shortening (Weng et al., 1995; Rufer et al., 1999) and cellular senescence over time. In line with this, after clonal selection *in vitro* leads to accumulation of replicatively senescent T-cells. In addition, senescence-like T-cells also occur *in vivo* (Effros, 2004).

In addition to telomere shortening, changes in the expression of several transcription factors also affect thymic involution (Trebilcock and Ponnappan, 1996; Ortman et al., 2002). Moreover, alterations in epigenetic regulation have also been associated with immunosenescence. Kuwahara et al. (2014) revealed that Menin promotes histone acetylation at the Bach2 locus, thereby suppressing T-cell senescence and ultimately immunosenescence (Kuwahara et al., 2014). Additionally, our laboratory showed that immunosenescence in rats is paralleled by substantial changes in thymus and spleen transcriptome of old rats. Additionally, aging is associated with a loss of heterochromatin marks, including H3K9me3 with corresponding reduction in SUV39H1 expression and increased genomic instability in the thymus (Sidler et al., 2013). This may contribute to the induction of senescence and apoptosis in cells within the aging thymus. However, further research is necessary to identify the role of epigenetic regulation in immunosenescence.

## DNA methylation and aging

Analysis of DNA methylation in the genomes from CD4^+^ T-cells from a newborn and a centenarian revealed a global loss of DNA methylation with age with differentially methylated sites identified in promoter (~10%), exonic (~10%), intronic (~45%), and intergenic (~35%) regions (Heyn et al., 2012). When Heyn et al. (2012) compared the global methylation levels in the centenarian and newborn genomes to the level in the genome of a 26-year-old, they found that the global DNA methylation level in the 26-year-old was intermediate. Thus, they proposed a model that asserts cells accumulate stepwise changes in DNA methylation throughout a lifespan.

Age-dependent decrease in global genome methylation has also been observed in other aging model organisms such as mice (Wilson et al., 1987) and rats (Vanyushin et al., 1973), although more recent studies do not always support these early findings. For example Maegawa et al. (2010) found that more cytosines became hypermethylated in older mice, as compared to those that became hypomethylated; analysis of methylation in 3,627 autosomal genes showed that 774 (21%) of them became hypermethylated, whereas only 466 (13%)—hypomethylated in 35-month vs. 3 month-old C57BL/6 mice (Maegawa et al., 2010). The inhibition of DNA methylation resulted in reduced replicative lifespan of cells (Holliday, 1986). However, the extent of global DNA methylation changes and the CpG sites affected may vary between different tissues. For example, DNA methylation levels in rat adipose tissues did not significantly change with age (Thompson et al., 2010).

While global DNA hypomethylation is observed in various tissues with age, the accumulation of domains of heterochromatin during the early steps leading to senescence has been described (Narita et al., 2003). These senescence-associated heterochromatin foci (SAHF) were originally shown to coincide with E2F target promoters. However, while pRB and p53 were demonstrated to be important for the establishment of SAHF, senescence in response to oncogene activation also involved the formation of SAHF (Ye et al., 2007). The observation that telomeres and subtelomeric regions became de-heterochromatinized in the absence of telomerase in aging mice (Benetti et al., 2007) led Zhang and Adams to propose that during aging, the heterochromatin marks get redistributed from areas of constitutive heterochromatin, such as telomeres and pericentromeres, to areas of facultative heterochromatin, such as SAHF (Zhang and Adams, 2007).

When analyzing the observed changes in DNA methylomes of human blood cells in more detail, Heyn et al. (2012) indicated DNA hypomethylation primarily occurred in CpG-poor promoters or tissue-specific promoters. It was also shown that hypomethylation is more frequent in the genomics regions enriched with polycomb proteins or permissive histone modifications (McClay et al., 2014). In contrast, DNA hypermethylation, was primarily found CpG-rich sequences. Interestingly, when we compared gene expression and DNA methylation patterns in WI38 cells throughout their lifespan, we observed a similar trend to DNA hypomethylation with some genomic sites also being hypermethylated, but also found that they rarely corresponded with differential gene expression (Sidler et al., 2014a).

These findings are further backed up by a mathematical modeling study performed on 82 available Illumina DNA methylation array data sets to test for the presence of age predictor sites in the genome (Horvath, 2013). Horvath has identified 353 differentially methylated CpG sites that predicted age very robustly, which has resulted in the proposition of an epigenetic clock model for aging. Among these 353 CpG sites 193 sites were commonly hypermethylated and 160 hypomethylated with increasing age. While the methylation status of the hypermethylated sites was quite robust across different tissues, the hypomethylated sites exhibited more tissue-specificity (Horvath, 2013). Also, while hypomethylated sites were more commonly found in CpG shores than hypermethylated sites, hypermethylated sites were over-represented near Polycomb-target genes. However, the overlap with gene expression changes associated with these age predictor sites was low, so it is not quite clear how the hypermethylation at these sites affects aging.

It was observed that DNA hypermethylation is more site-specific as compared to hypomethylation. Importance of DNA hypomethylation for triggering senescence is demonstrated by two facts. First, hypomethylation occurs in senescing cells but not in immortalized cells (Wilson and Jones, 1983; Degerman et al., 2014). Second, DNA methylation inhibition triggers growth arrest in immortal cells (Vogt et al., 1998).

As a result, the shift to hypomethylation of constitutive heterochromatin and hypermethylation of promoters in cell-cycle promoting genes, age-dependent changes in DNA methylation may trigger increased genomic instability and lead to a permanent cell-cycle arrest. Furthermore, the differential methylation of promoter CpGs may also contribute to the observed changes in senescence- and age-related gene expression profiles. The role of DNA hypomethylation and site-specific hypermethylation in aging and senescence would have to be clarified in the future research.

## Post-translational histone modifications and nucleosome occupancy and aging

Post-translational modifications of the histone amino tails affect their affinity to DNA and interacting proteins, and different combinations of such modification marks may have synergistic or antagonistic effects on those affinities. This indicates a role of histone modifications in formation of active or repressive chromatin states and led Jenuwein and Allis to the formulation of the “histone code” theory (Jenuwein and Allis, 2001). Generally, acetylation and phosphorylation of histone tails are considered to be euchromatin marks and methylation of histones is enriched in heterochromatin regions (Jenuwein, 2001). However, some exceptions to this theory exist. For example, H4K20 acetylation is a repressive mark (Kaimori et al., 2016), whereas H3K4 and H3R17 methylation are activating marks. The effect of histone modification marks is mediated by bromodomain-containing proteins in the case of acetylation marks (Dhalluin et al., 1999) or by chromodomain-containing proteins in the case of methylation marks (Bannister et al., 2001).

In addition to histone tail modifications, the nucleosome density affects the level of DNA packaging as well. Therefore, DNA regions with a high density of nucleosomes are likely transcriptionally inactive while transcriptionally active DNA regions are characterized by looser packaging and a low density of nucleosomes (Boeger et al., 2003).

Both nucleosome occupancy and histone tail modifications experience changes as cells age (Table 1). A decline in nucleosome occupancy with increasing age was first demonstrated in human skin fibroblasts in which the linker regions between nucleosomes were shown to become increasingly heterogeneous in length (Ishimi et al., 1987). The global loss of histones has been hypothesized to be associated with a reduced deposition of histones, which is mediated by ASF1 in a replication-dependent and a replication-independent manner together with CAF1 and HIRA (Groth et al., 2007; Galvani et al., 2008), or by reduced expression, also regulated by ASF1 during replication (Sutton et al., 2001), or the maturation histone mRNA promoted by SLBP (Kaygun and Marzluff, 2005). The loss of function of ASF1 in yeast resulted in impaired heterochromatin formation and genomic instability (Tanae et al., 2012). The finding that ASF1 and SLBP expression decrease with age in human cells (O'Sullivan et al., 2010) and that over-expression of histones increases lifespan (Feser et al., 2010), may indicate that declining nucleosome occupancy in aging human cells similarly results in a loss of heterochromatin paired with increased genomic instability. This also likely results in changes to the gene expression profile. However, to better understand the potential role of nucleosome loss in aging, a more detailed understanding of DNA regions affected by a reduced nucleosome density and the functional consequences is needed.

**Table 1:** Changes in histone modification pattern and nucleosome occupancy with increasing age and senescence.

**Modification**	**Yeast**	**Mouse**	**Rat**	**Human**	**References**
Global acetylation				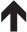	Ryan and Cristofalo, 1972
H4 acetylated			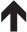		Pina et al., 1988
H3K9me				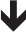	O'Sullivan et al., 2010
H3K9me2				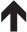	O'Sullivan et al., 2010
H3K9me3		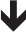		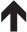 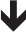	Narita et al., 2003; Braig et al., 2005; Scaffidi and Misteli, 2006; O'Sullivan et al., 2010; Di Micco et al., 2011; Larson et al., 2012
H3K9ac		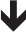	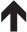	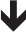	Mostoslavsky et al., 2006; Scaffidi and Misteli, 2006; Michishita et al., 2008; Kawakami et al., 2009; O'Sullivan et al., 2010
H3S10phospho			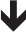	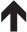	Kawakami et al., 2009; O'Sullivan et al., 2010
H3K14ac		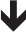			Huang et al., 2007
H3K27me3		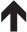		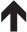	Bracken et al., 2007; Dhawan et al., 2009
H4K8ac		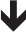			Huang et al., 2007
H4K12ac		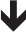			Huang et al., 2007; Peleg et al., 2010
H3K56ac	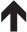			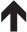	Dang et al., 2009; Feser et al., 2010; O'Sullivan et al., 2010
H4K16ac	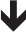	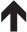		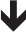 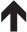	Dang et al., 2009; O'Sullivan et al., 2010
H4K20me				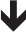	O'Sullivan et al., 2010
H4K20me2				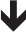	Sarg et al., 2002; O'Sullivan et al., 2010
H4K20me3			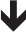	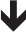 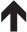	Sarg et al., 2002; O'Sullivan et al., 2010
H3.1	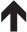		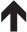	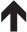	Pina and Suau, 1985; Rogakou and Sekeri-Pataryas, 1999; Feser et al., 2010; O'Sullivan et al., 2010
H3.2	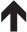		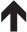	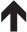	Pina and Suau, 1985; Rogakou and Sekeri-Pataryas, 1999; Feser et al., 2010; O'Sullivan et al., 2010
H3.3			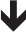	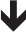	Pina and Suau, 1985; Rogakou and Sekeri-Pataryas, 1999
H4				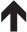	O'Sullivan et al., 2010
H2A.1	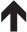		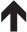	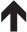	Pina and Suau, 1985; Rogakou and Sekeri-Pataryas, 1999; Feser et al., 2010
H2A.2			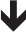	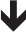	Pina and Suau, 1985; Rogakou and Sekeri-Pataryas, 1999
yH2AX		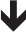		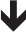	d'Adda di Fagagna et al., 2003; O'Sullivan et al., 2010

*Arrows indicate age-related up- (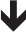) or down-regulation (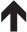)*.

In addition to overall changes in nucleosome numbers, numerous changes in post-translational modifications of histone tails with age have been described (Table 1). However, their functional consequences are only beginning to be understood. H3K56ac promotes nucleosome assembly and transcriptional activation of histone gene expression (Kaplan et al., 2008; Williams et al., 2008). H3K9me3 is involved in heterochromatin formation in telomeric, subtelomeric, and pericentromeric regions in young cells (Benetti et al., 2007; Vaquero et al., 2007) and with the establishment of SAHF in senescing cells (Narita et al., 2003). Finally, H4K16ac is involved in telomere silencing (Kozak et al., 2010).

Aforementioned histone marks are established by various histone-modifying enzymes including histone deacetylases and acetyltransferases (Kuo and Allis, 1998), histone methyltransferases, and histone demethylases (Black et al., 2012). Thus, changes in the frequencies of different histone modifications may be associated with different expression or activity levels of histone-modifying enzymes. In line with this, the redistribution of SIRT1 from heterochromatin regions to sites of DNA damage resulted in gene expression changes resembling those in aging tissues (Oberdoerffer et al., 2008). The importance of SIRT1, a histone deacetylase, in the processes of normal cell proliferation and aging cannot be underestimated. SIRT1 deacetylates and inactivates the RelA/p65 subunit of transcription factor NF-κB known to be the main transcriptional regulator of several genes directly involved in inflammation processes associated with aging (Lawrence, 2009).

Moreover, SUV39H1 seems to play an important role in controlling the proliferative status of a cell, through alterations of the chromatin structure at centromere region during the process of cell division (Aagaard et al., 2000), the organization of the nuclear architecture (Uhlirova et al., 2010), the inhibition of cell differentiation in transgenic mice overexpressing SUV39H1 (Czvitkovich et al., 2001), and the reduction of viability along with increased genomic instability and susceptibility to cancer found in SUV39H1/2 knockout mice (Peters et al., 2001).

Furthermore, reduced SUV39H1 levels during cell senescence and aging (Sidler et al., 2013, 2014b) are associated with reduced H3K9 trimethylation in pericentric satellite regions. Since the relaxation of satellite heterochromatin has been shown to occur early during the establishment of senescence (Swanson et al., 2013), these observations may indicate that SUV39H1 plays an important role in promoting genome stability through its role in heterochromatin formation, thereby allowing for continued cell division.

Additionally, SUV39H1 is involved in regulating gene expression. For instance, SUV39H1 cooperates with E2F/RB to silence S-phase genes in terminally differentiating cells (Ait-Si-Ali et al., 2004), and contributes to transcriptional repression of the p21 promoter (Cherrier et al., 2009). During senescence, SUV39H1 is either enriched or reduced at specific promoters. Such specific recruitment of SUV39H1 by transcription factors may be essential for modulation of H3K9me3 patterns during aging (Ait-Si-Ali et al., 2004; Lomberk et al., 2012), and may in addition segregate SUV39H1 away from constitutive heterochromatin regions.

Studies of the effects of SUV39H1 mutations on lifespan are complicated as both the overexpression and the knockout lead to developmental defects (Czvitkovich et al., 2001; Peters et al., 2001). However, one study showed that mice deficient in Zmpste24 and Suv39h1 lived 11 weeks longer than mice deficient for Zmpste24 alone (Liu et al., 2013). However, the reason for the lifespan extension by Suv39h1 deficiency in this background may also be related to its role in promoting heterochromatin DNA repair (Zheng et al., 2014) rather than its role in cell proliferation, as Zmpste24 deficiency is known to lead to accumulation of damaged and unrepaired DNA (comparable to senescent cells) (Liu et al., 2005). It is quite likely that during the establishment of cellular senescence the downregulation of SUV39H1 may initially occur to promote DNA repair leading to genome destabilization due to deheterochromatinization of repetitive DNA.

To sum up, the downregulation of SUV39H1 during the establishment of senescence may promote DNA repair in heterochromatin regions but eventually results in increased genomic instability promoting cell-cycle arrest. On the other hand, the altered expression and recruitment of SUV39H1 to promoter regions of genes may also shape the senescence-associated gene expression profile and further contribute to the establishment of senescence. Further research is needed to clearly define the regulation and role of SUV39H1 during the establishment of senescence.

## Future directions

### Role of epigenetic senescence mechanisms in stem cells and cancer

Both SUV39H1/2 knock-out mice and SUV39H1 transgenic mice showed severe developmental defects, while the knockout animals exhibited decreased viability, increased genomic instability, and susceptibility to tumor formation (Peters et al., 2001). The transgenic animals overexpressing SUV39H1 showed a deficiency in cell differentiation (Czvitkovich et al., 2001). Our results further support a role for the SUV39H1 downregulation in the induction of senescence (Sidler et al., 2014b). Being a histone methyltransferase, SUV39H1 expression is important for the maintenance of adequate levels of H3K9 trimethylation in order to silence pericentric repetitive DNA regions (Peters et al., 2001; Lehnertz et al., 2003) and to regulate transcriptional repression through increased H3K9me3 levels or transcriptional activation through the reduction in H3K9me3 levels.

Thus, it can be hypothesized that adequate SUV39H1 expression levels are required in order to balance genomic stability and proliferative potential. In such a model, increased SUV39H1 expression levels would protect the genome integrity and promote cell division, whereas decreased SUV39H1 expression levels would increase genomic instability and favor cell cycle arrest; however, if the SUV39H1 expression levels fall below a threshold level, the silencing of promoter regions of growth inhibitory genes may also be compromised and therefore result in tumor formation. In addition to mere differences in expression levels, the altered recruitment of SUV39H1 to specific DNA regions likely also contributes to senescence-associated changes in the distribution of H3K9me3 marks. In order to test this model for the role of SUV39H1 expression and recruitment in balancing genomic integrity and cell proliferation, it would be interesting to address the following questions.

While the role of SUV39H1 in the formation of the heterochromatin in pericentric regions is well-accepted, the patterns of SUV39H1-mediated histone H3K9 trimethylation in other DNA sequence regions are less characterized. A better understanding of which transcription factors interact with SUV39H1 and may therefore recruit it to their target promoters could help researchers to understand the effects of SUV39H1-mediated H3K9 trimethylation on gene expression. One such example is the association of SUV39H1 with RB/HDAC1 resulting in the heterochromatin formation and suppression of genes involved in cell cycle progression (Bandyopadhyay et al., 2007). Thus, a systematic analysis of the interactome of SUV39H1 and ChIP-seq determination of the target sequences of SUV39H1-mediated heterochromatin formation in dividing when compared to senescent cells will allow researchers to further dissect the role of SUV39H1 in senescence-associated changes in transcriptional regulation and the role of SUV39H1 recruitment in the establishment of senescence.

In addition to mediating the silencing of repetitive DNA regions, SUV39H1 also plays a significant role in the regulation of telomere length (Garcia-Cao et al., 2004), and telomere nuclear architecture (Uhlirova et al., 2010). Furthermore, heterochromatin regions enriched with repressive histone marks associate with the nuclear lamina (Guelen et al., 2008) and likely contribute to the domain organization of chromosomes, with regions more distant to the nuclear lamina being more frequently transcriptionally active (Peric-Hupkes et al., 2010). Peric-Hupkes et al. (2010) showed that this domain organization changed with increasing differentiation of mouse embryonic fibroblasts, resulting in differential gene expression. Further, a study by Zhang et al. (2015) showed that upon inducing WRN deficiency in mesenchymal stem cells, the cells exhibited various aging phenotypes, including a global loss of H3K9me3 and changes in heterochromatin architecture (PMID: 25931448). The others also showed that expression of catalytically inactive SUV39H1 in wild-type stem cells resulted in a similar phenotype, further supporting a role for SUV39H1 in preventing senescence. Hence, it would be interesting to test whether the downregulation of SUV39H1 during the establishment of senescence corresponds with alterations to the nuclear architecture and chromosome domain organization. If so, further testing could show whether this results in altered gene expression patterns that might contribute to the establishment of senescence.

In order to address how gradual changes in SUV39H1 expression levels affect the cell-cycle dynamics, human diploid fibroblasts could be treated with increasing amounts of SUV39H1 targeting siRNA or transfected with increasing amounts of an SUV39H1 overexpression construct followed by analysis of cell proliferation by BrdU incorporation and senescence by the SA-beta-GAL assay. This would allow for testing of the hypotheses that have been formulated above and might help in determining the threshold of the SUV39H1 expression level that is necessary for the inhibition of cell division if such a threshold indeed exists.

Another approach is to determine how the SUV39H1 expression levels correlate with the rate of proliferation in various cell types. For this, it would be interesting to compare various cancer cell lines, stem cells, and human diploid fibroblasts at different population doublings and primary cells derived from donors of different ages.

A more detailed understanding of how SUV39H1 expression is controlled on the level of transcription and post-transcriptionally will help in studying what intra- and extra-cellular stimuli may trigger the downregulation of SUV39H1. Two studies have described an indirect role of p53 in regulating the expression of SUV39H1 by inducing the transcription of p21, which in turn represses E2F mediated transcription of SUV39H1 (Mungamuri et al., 2012; Zheng et al., 2014). In addition, miRNA-mediated post-transcriptional regulation of SUV39H1 expression has also been described (Villeneuve et al., 2010). A systematic promoter analysis of the SUV39H1 promoter region, as well as a target screen for miRNA-mediated post-transcriptional regulation, may indicate additional regulatory mechanisms that may affect the SUV39H1 expression during cell differentiation and cell proliferation. In addition, our study of the treatment of cells with chaetocin resulted in the reduction of SUV39H1 transcript levels (Sidler et al., 2014b), which was surprising because chaetocin inhibits the enzymatic activity of SUV39H1. This may therefore indicate that SUV39H1 expression is also regulated by a positive feedback mechanism. Therefore, it would be important to find out whether this is the case and how it is mediated. One possibility for such a mechanism could be that SUV39H1 may methylate one of its own transcriptional repressors and thereby inactivate it.

### Role of differential DNA methylation in senescence

Several studies have described altered DNA methylomes that were characterized by extensive site-specific DNA hypomethylation alongside of some site-specific hypermethylation with increasing age (Heyn et al., 2012; Johansson et al., 2013; McClay et al., 2014). We made similar observations in senescence when compared to actively dividing cell cultures of WI38 human lung fibroblasts (Sidler et al., 2014a). While our dataset showed that there were some clusters of DNA hypo- or hyper-methylation throughout the genome, differential methylation of promoter regions rarely corresponded with a change in gene expression. In addition, the global loss of DNA methylation and downregulation of DNMT1 correlates with the observation that the majority of differentially methylated CpG sites in senescent cells are hypomethylated. However, it will be interesting to further address the role of this altered DNA methylome in senescence and whether it is a consequence of other senescence-related molecular changes or otherwise plays a causal role in the establishment of senescence. Furthermore, it would be interesting to study how certain sites are targeted for hypo- or hyper-methylation.

The downregulation of DNMT1 during senescence could indicate that DNA methylation marks are passively lost through the deficiency in maintenance through cell divisions. However, 1,849 CpG sites were significantly differentially methylated when comparing three senescent to three dividing cell cultures in our study, pointing that the loss of DNA methylation during senescence may at least in part be non-random. Thus, it would be interesting to study whether the recruitment of DNMT1 to DNA is altered during senescence. This could be achieved through the immunoprecipitation of DNMT1 followed by mass-spectrometric analysis of its interacting proteins in order to determine whether DNMT1 is associated with different binding partners during senescence. Some studies suggest a role for the existing histone modification pattern in limiting the *de novo* DNA methylation in a sequence-specific way (Schlesinger et al., 2007; Straussman et al., 2009; Ludwig et al., 2014). Consequently, a better understanding of the regulation of the histone modification pattern during senescence may help in answering this question.

Changes in the DNA methylation patterns do not extensively correlate with altered gene expression, but the inhibition of DNA methylation was shown to trigger cell growth arrest (Vogt et al., 1998) suggesting that DNA hypomethylation is associated with the molecular changes that inhibit cell division. This could potentially be mediated through contributing to genomic instability through the loss of heterochromatin or to the altered nuclear architecture potentially by affecting the nuclear lamina interaction and chromosomal domain organization (Guelen et al., 2008) and could be studied with similar approaches described for SUV39H1 above.

### The role of DNA methylation and histone modifications in immunosenescence

In order to dissect the role of epigenetic changes in thymic involution further, it would be interesting to separate the different cell types that occur within the thymus, dendritic cells, epithelial cells, nurse cells, macrophages, and maturing T-cells. The measurement of DNMT1 and SUV39H1 protein levels and determination of global DNA methylation and H3K9 trimethylation in the different cell types could then indicate which cells are mainly affected by the loss of heterochromatin marks and corresponding increase in genomic instability.

Furthermore, our study involved only three time points across the lifespan of the rats—1month-old animals, 4 month-old animals around the age of the onset of the thymic involution, and 18-month-old animals, which have a very small thymus (Sidler et al., 2013). The analysis of additional time points around the onset of thymic involution may help in determining cell type and the stage of thymus development in which the downregulation of SUV39H1 and DNMT1 occurs. If it occurs relatively early, this may suggest that the associated heterochromatin loss, genomic instability, and alterations in the gene expression profile may play a causal role in the induction of senescence and/or apoptosis in the affected cell types. If it occurs relatively late, it may rather be a consequence of other age-related changes within the thymus, such as DNA damage-induced downregulation of SUV39H1 in order to facilitate the repair of DNA damage within heterochromatin regions.

If the downregulation of SUV39H1 and DNMT1 does occur relatively early during thymic involution, it would be interesting to further study their role in the onset of thymic involution by creating mouse-models that specifically overexpress or repress SUV39H1 or DNMT1 in the thymus or possibly even in a specific cell type that is affected by their age-dependent downregulation (Sidler et al., 2013). The phenotype of such mouse-models would give further indications on what the specific role of the SUV39H1- and DNMT1-downregulation is during thymic involution.

On the other hand, since the proliferation of peripheral T-cells depends on antigen recognition, which is limited in the rats we studied as they were housed in a pathogen-free environment (Sidler et al., 2013), it would be interesting to study epigenetic changes in T-cell populations that are challenged with pathogens. This could be achieved either *in vivo*, through the repeated infection with a virus, such as CMV, followed by collection of blood samples in order to study the T-cell population that was clonally selected in response to each cycle of infection, or *in vitro*, by exposing virus-specific T-cells to their cognate antigen for several subcultures and studying changes in epigenetic regulation.

## Conclusions

Considerable progress has been made in understanding regulatory pathways that are involved in aging over the recent years. Several studies have described age-dependent reduction in global and constitutive heterochromatin affecting both DNA methylation and histone modifications as well as site-specific changes in DNA methylation and histone modification patterns. A more detailed understanding of the regulation of the modifying enzymes and their recruitment during the establishment of senescence will likely pinpoint several molecular targets that are involved in the regulation of aging and may even serve as possible drug targets to promote a prolonged, healthy lifespan.

## Author contributions

CS: Wrote the manuscript; OK: Proposed the topic, revised the manuscript; IK: Proposed the topic, wrote the manuscript.

### Conflict of interest statement

The authors declare that the research was conducted in the absence of any commercial or financial relationships that could be construed as a potential conflict of interest.
