# Supplementary material for: Epigenetic Regulation of Cellular Senescence and Aging
Source: Front Genet. 2017 Sep 26;8:138. doi: 10.3389/fgene.2017.00138 (PMC5622920; doi:10.3389/fgene.2017.00138)
Supplement: Supplementary file 1 [file Table1.DOCX]

Table S1. Effects of gene mutations, caloric restriction and telomere length on lifespan extension in aging model organisms

This table shows genes that when mutated extend organismal lifespan. Additionally effects of caloric restriction and telomere length on lifespan extension are also indicated.

|  | **Yeast** | ***Drosophila*** | ***C. elegans*** | **Mouse** | **Rat** | **Human** |
| --- | --- | --- | --- | --- | --- | --- |
| **Caloric restriction** | [1] |  | [2] | [3-5]^*^ | [6] | [7] ^1^ |
| **Insulin signalling** | Glucose signalling: *CDC25*, *TPK1,* *TPK2, TPK3, HXK2, GPR1, GPA2* [8] | *InR* [9]; *chico* [10]; *Lnk* [11] | *daf-2* [12]; *age-1* [13]; *oga-1* [14]; *pdk-1* [15]; *akt-1* ^ ^[16, 17] ^,6^; *akt-2* [17] | *Igf1r* [18, 19]^3^; *Papp-a* [20]; *Irs1* [21]; *Akt1* [22] ^^ |  | *IGF-1R* [23] ^4^ |
| **Hormones** |  | *EcR* [24] ^3^ | *daf-12* [25]^*^; *nhr-154, nhr-14* [16] ^^ | *Ghrh* [26]; *Ghrhr* [27]; *Ghr* [28] | *Gh* [29] | *PPARG* [30] ^4,*^ |
| **TOR signalling** | *TOR1* [31, 32]; *GLN3, LYS12, MEP3, AGP1, MEP2* [33]; *SCH9* [34] | *dTOR, dS6K* [35] ^5^ | *rsks-1* [36, 37] ^,6^; *let-363/CeTor* [38]^6^; *daf-15/raptor* [39]^3^; *sinh-1* [40]^2^ | *mTor* [41]; *S6k1* [42] |  |  |
| **Translation** | *RPL31A, RPL6B* [31, 43]; *RPL9A, RPL19A* [32, 43]; *TIF4631* [32, 44]; *TIF1, TIF2* [32, 45]^*^; *RPL20B* [43, 44]; *RPL23A, RPL34B, RPL21B, RPP2B, RPL22A, RPL43B, RPL13A, RPL7A, RPL29* [43]; *RPL10, RPS18A, RPS18B, RPS6B* [46]; *ELP4, TMA19, RPL37B* [44] |  | *ife-2* [36, 47]^6^; *rps-15* [36, 48]^2^; *rpl-6* [36]^2^; *ifg-1* [36, 37]^2^; *eIF2Bδ* [49]^2^; *iftb-1* [36]^2^; *rpl-28, rpl-P2, aars-2* [50]^2^; *rpl-17* [48, 50]^^; *iff-1* [16]^2^; *drr-2* [40]^2^; *Y39G10AR.8, D2085.3, eif-3.F, nol-5, rpl-19, nars-1, Y48G1A.4, tars-1* [51]^2^; *mars-1, F33D4.5* [52]^2^; *rps-23* [48, 52]^2^; *rps-5* [48, 52]^2^; *rps-16, inf-1, rpl-8, eef-1A.1, rps-0, eef-2, rla-0, rpl-23, rps-20, rpl-31, rpl-10a, rps-14, rars-1, rpl-10, rpl-7, rps-11, rpl-3* [48]^2^; *gro-1* [53] |  |  |  |
| **Ubiquitin ligation/ proteolysis** | *UBR2, MUB1* [54]; *BRE5* [31]; *TOM1* [44]; *AFG3, HSE1* [32] |  | *vhl-1* [55]^6^; *ubh-4, rnf-5, egl-3, nas-38, Y71H2AR.2* [16]^2^; *Y71H2_385.b, F49C12.9* [52]^2^; *rle-1* [56] |  |  |  |
| **Epigenetics** | *YTA7, HMT1* [45]; *SAS2* [57]; *SAM1* [32]; *SET2* [58]; *HHF1* [59] | *hop* [60]^3^ | *ash-2, wdr-5, set-2, set-4* [61]^6^; *rbr-2* [61-63]^6,*^; *set-26, set-9, mes-2, jmjd-2* [62]^2^; *lsd-1,* *jhdm-1* [63]^2,3,6^;*,* *utx-1* [62, 63]^2,3,6^; *sams-1* [40]^2^; *epc-1* [52]^2^; *his-37* [48]^2^; *miR-80* [64]; *set-15, rbbp-5, cec-3* [16]^2^; *let-418* [65] |  |  | *MIR-21* [66]^1^ |
| **Transcriptional regulation / transcript processing** | *SPT4, DBP3, TIS11* [32]; *GCN4, LOC1, SWI5* [44]; *FZF1* [67]; *LEU3* [68]; *UGA3* [67, 69]; *URE2* [31] |  | *lin-14* [70]^6^; *ets-4* [71]; *spr-3* [72]^6,*^; *hif-1* [55, 73, 74]^6,*^; *spt-4, ceh-18, pup-2* [16]^2^; *rha-2* [40]^2^; *C48E7.2, rpoa-2* [51]^2^; *F08B4.7* [52]^2^; *elt-6* [52, 75]^2^; *elt-5* [75]^2^; *crtc-1, tax-6* [76]^2^; *hcf-1* [77]^6^; *exos-3* [51]^2^; *car-1, snr-2, cgh-1, pgl-1* [50]^2^; *pab-1* [48, 50]^2^ | *Pit1 (=Pou1f1)* [27]; *Prop1* [78] |  |  |
| **Cell cycle regulation / control of senescence** | *MAD2, PCH2* [45] | *Dmp53* [79]^5^ | *cid-1, chk-1, cdc-25* [80] ^6^ | *p16ink4a, p19arf* [81]^1,2^; *Cables* [82] ^1^ |  | *p53* [83-86]^1,3,5^; *p16INK4A* [87, 88]^1,2^; *p21, RB* [86]^1,3^; *BTG2* [89]^1,2^;  *CHK2* [90]^1,5^ |
| **Oxidative stress** | *PDX3* [45]; *CTA1* [91] |  | *sod-2* [92]; *F53H1.3, T12E12.1, C03H5.3, lbp-6* [93]^2^ | *p66Shc* [94] |  |  |
| **Mitochondrial dysfunction** | *MRG19* [95]; *AFO1* [96]; *SOY1* [59]; *RIM1* [44]; *MSW1* [44, 45]^*^; *YBR238C* [31] | *sbo* [97]^3^ | *frh-1* [98, 99] ^2,*^; *clk-1* [53, 100]; *nuo-6* [101]^6^; *isp-1* [101, 102]^6^; *mfn-1* [103]^2^; *lars-2, F13G3.7, K01C8.7, F28B3.5* [104]^2,6^; *cchl-1* [40, 104]^2^; *D2030.4, F26E4.6* [16, 104]^2^; *W09C5.8* [16, 104, 105]^2^; *cco-1* [16, 40, 104-106]^2^; *C33F10.12, asg-2* [16]^2^; *T20H4.5* [16, 52]^2^; *nuo-2* [40, 106]^2^; *nuo-5, atp-4, atp-5, asb-2* [40]^2^; *nuo-4* [16, 40, 52]^2^; *lpd-5, F26E4.4, F59C6.5, sco-1, R53.4,* *mrpl-24, mrps-30, spg-7, mrpl-10* [51]^2^; *tag-99, mrps-9, mrpl-12, Y56A3A19, mrps-10, ant-1.1, Y119D3_463.b, Y53G8A_9248.c, Y53G8A_9248.d, Y71H2_378.a, Y71H2_388.d, year-1, cyc-2.1, cyp-33E2, mrps-33, ZK809.3, C47E12.2, gars-5, Y55F3B_743.b* [52]^2^; *F29C4.2* [52, 105]^2^; *nuo-3* [40, 52, 105]^2^; *C34B2.6, dlat-1, F01G4.6, B0250.5* [48]^2^; *ucr-1* [48, 52, 105]^2^; *mrps-5, nkcc-1, ttll-9, mrpl-1, mrpl-2, mrpl-37* [107]^2^; *nuo-1* [105, 108]^2,6^; *atp-2* [48, 108]^2,6^; *C33A12.1* [52, 105]^2^; *C25H3.9, C18E9.4, ucr-2.3, F45H10.2, R07E4.3, T27E9.2, Y71H2AM.5, cco-3, cco-4* [105]^2^; *tag-174* [52, 105]^2^; *cco-2* [40, 105]^2^; *phi-44* [104, 105]^2^; *cyc-1* [40, 105, 106]^2^; *atp-3* [40, 106]^2^; *hsp-6* [109]^2^; *sft-1, oxa-1* [110]^2^; *mrpl-47* [16, 104]^2^; *mics-1, atad-3* [111] ^6^ | *Mclk1 (Coq7)* [112]^3^; *Surf1* [113] |  | *UCP1* [114] ^4^ |
| **DNA repair / genome integrity** | *RNH201* [45]; *FOB1* [31] |  | *clk-2* [53] |  |  |  |
| **Telomere integrity/length** | *TLC1* [115]^^ |  | Long telomeres [116] |  |  | Long telomeres [117] ^1^ |
| **Other functions** | *CYS4, DNF1, UBC12, PRS3,* *ALD4, PHO89, DIE2, ALG5, SSA3, LSB6* [45]; *GAD1* [69]; *UGA1* [45, 69]; *REI1, ROM2, MTC4, SLM6, IRC14* [31]; *IDH2* [31, 32]; *ADH1, IDH1, PMR1, SIS2, YGR130C, YPT6, INP53* [32]; *INP53* [32, 45]^*^; *SOK1, SPS1, BOI2,* *SIP2, SNF1* [44]; *INP51* [32, 44]; *URH1* [67]; *GUP1, HXT17, IPK1* [59]; *PKH2* [32, 59]; *CYR1* [8, 118]; *BST1, ERO1, EUG1, MPD1, HRD1, DER1, ALG3, OST3* *ALG12* [32, 119] | *snz* [120]; *Dacer* [121]; *ECP* [122]^2^; *Indy* [123, 124]^*^; *mth* [125]; *loco* [126]^3^; *ilk, mys* [127]^3^ | *fgt-1a, fgt-1b* [128]^2^; *odr-2, odr-3, odr-7* [129]; *inx-14, pfn-2, K07H8.1, col-93, cdh-12, C35A11.3, clec-227, cwp-4, clec-186, T05A1.4, D1054.8, mecr-1, K08E3.5, idha-1, idh-1, F55B11.1, pghm-1, unc-83, nrfl-1, trim-9, F09F7.5, Y39H10A.6, Y54E5A.7, C36H8.1, scl-4, Y39F10C.1, cpna-3, gcy-29, C27B7.7, max-1, F35D2.3, scrm-8, K10B4.3, sru-17, srw-20, srh-254, F40F8.5, C09B7.2, C26B2.2, C32H11.1, E03H12.5, F49F1.12, H06H21.8, R05A10.5, R08E3.3, T06G6.4, T07A9.8, Y43F8B.12, laat-1, Y46H3C.6, Y53F4B.23, Y56A3A.9, Y75B8A.13, Y75B8A.33, sid-2* [16]^2^; *ttr-1, maoc-1, gpi-1, ddl-1, ddl2, ddl-3, pat-6, ril-1, ril-2, rab-10* [40]^2^; *drr-1* [16, 40]^2^; *K11B4.1, abcx-1, B0491.5* [51]^2^; *pat-4* [40, 52, 130]^2^; *ral-1, Y71H2_388.ct, hhat-2, asm-3, Y69A2A_2991.c, Y43F4B.7, Y119D3_446.d, Y53G8A_1734.g, grl-19, F42A6.1, aat-8, T05A1.5, amt-2, scl-8, cutl-28, tba-7,* *Y71H2_390.d, tes-1, F56D5.5, inx-8, inx-9, W03G1.5, acs-5, ogdh-1, acdh-13, tkt-1, dld-1, K12H4.5, T28D6.4, pod-1, B0546.3, spe-26, Y45F10D.8, ttr-5, Y66A7A1, T28A8.6, Y39A3C_82.a, Y53G8A_2702.a, Y53G8A_9248.b, F09C11.1, W07G9.1, C46G7.2, F13B6.1, K07H8.8, R10H10.7, C42C1.3, Y51H4A.m* [52]^2^; *pyc-1, mdh-1, tbb-2, F09F7.4, gdh-1, cpn-3, cct-1, acs-4, rack-1, cct-8* [48]^2^; *sca-1* [48, 131]^^; *ser-1, tph-1* [132]; *sea-2* [133]; *tatn-1* [134]^6^; *eak-7* [17, 134]^6^; *slcf-1* [135]^6^; *glp-1* [17]; *clk-3* [53]; *ipgm-1* [16, 104]^2^; *aco-2* [16, 48]^2^; *ndg-4, nrf-5, nrf-6, nrf-3* [136]; *ftn-1* [137]^2^; *arr-1, mpz-1* [138]^6^; *ocr-2, unc-31* [139]; *npp-16* [50]^2^ | *Adcy5* [140]; *RIIbeta* [141]^*^; *RasGrf1* [142] |  | *NUAK1* [143]^1,2^; *ADRB2* [144]^4^; *NOTCH3* [145]^1,2^; *MKP2, ERK2* [146] ^1,2,5^ |

^*^ lifespan extension, shortening or no effects on lifespan have all been described, ^1^ studied in a cell culture model, ^2^ RNAi experiment, ^3^ animals heterozygous for the mutation were studied, ^4^ polymorphisms over-/underrepresented among long-lived populations, ^5^ over-expression of dominant-negative form, ^6^ RNAi and genetic mutant studied, ^7^ gain-of-function mutant

Table S.2. Effects of gene mutations, caloric restriction and telomere length on lifespan shortening in aging model organisms

This table lists gene mutations, as well as caloric restriction and telomere length changes, that negatively affect organismal lifespan.

|  | **Yeast** | ***Drosophila*** | ***C. elegans*** | **Mouse** | **Rat** | **Human** |
| --- | --- | --- | --- | --- | --- | --- |
| **Caloric restriction** |  |  |  | [3-5]^*^ |  |  |
| **Insulin signalling** | Glucose signalling: *PDE2* [8] | *foxo* [10] | *daf-18* [147]; *ogt-1* [14] | *Irs2* [21]; *Socs2* [148] |  | *FOXO3A* [149-151]^4,*^ |
| **Hormones** |  |  | *daf-12* [25]^*^; *CeKlotho* [152] ^^ | *Klotho* [153]; *Fgf23* [154] |  |  |
| **TOR signalling** |  |  | *rict-1* [155] |  |  |  |
| **Translation** | *TIF1, TIF2* [32, 45]^*^; *RPL37A, SSF1* [45] | *Aats-met* [156] |  |  |  |  |
| **Ubiquitin ligation/ proteolysis** | *RPT1S, RPT2RF, RPT3R, RPT5R, RPT6R, UMP1, UBP6, PRE9* [54]; *UBC8, HRT3* [45] | *Uba1* [157] | *pbs-5* [50]^2^; *aip-1* [158]^2^; *F42G10.1* [131] ^2^ | *Topors* [159]; *Herc1* [160] |  | *WWP1* [161]^1,2^; *SENP1* [162] ^1,2^ |
| **Autophagy** | *ATG1, ATG7* [68]; *ATG18* [45] | *Atg7* [163] | *atg-7, lgg-3* [164]^2^; *atg-3, atg-4.1, atg-4.2, atg-5, lgg-1, atg-18* [165]^2^; *bec-1* [165, 166] ^6^ |  |  | *ATG7, ATG12, LAMP2* [167] ^1,2^ |
| **Sirtuins** | *SIR2* [168]; *SIR4* [168, 169]; *SIR3* [168] |  |  | *Sirt6* [170, 171] |  |  |
| **Epigenetics** | *GCN5, HAT1, CHD1* [45]; *NSI1* [172]; *RTT109, HST3, HST4* [57]; *DOT1, SET1* [58]; *BRE1* [173] | *miR-34* [174]; *Stat92E,* *Su(var)205* [60] ^3^; *mir-14* [175] | *rbr-2* [61-63]^6,*^; *lin-4* [70]^6^; *rde-4* [15]; *zfp-1* [15, 131]^2^ | *Cbx7* [176]^1^ |  | DNA hypomethylation [177-179]; *CBX7* 🡻 ^1^ [176] |
| **Transcriptional regulation / transcript processing** | *RBP4, RBP9, PAT1, DHH1* [180]; *RPN4* [54]; *NPL3, MRT4, SSN3* [45]; *SFP1* [96] |  | *nfi-1* [181]; *spr-3* [72]^6,*^; *skn-1* [182]; *ttx-3, ceh-10* [183]; *ire-1* [73]; *ldb-1,* *sup-37* [131]^2^ | *Clock* [184]; *Bmal1* [185] |  | *NRF2* [186]^1,2^; *NF1B* [66]^1^ |
| **Cell cycle regulation / control of senescence** | *CLB1, CLB2* [45] | *D-p35* [187]; *CycD, Cdk4* [188] |  | *BubR1* [189] |  | *CDC25A* [66]^1^ |
| **Oxidative stress** | *SOD1* [190, 191]; *SOD2, CCS1* [191]; *CTT-1* [192]; *TRX1* [45] | *cSOD* [193]; *Trx-2* [194, 195]; *GLaz* [196] | *trx-1* [197]; *glod-4* [198]^2^ | *MsrA* [199]; *Sod2* [200] |  | *TRX1* [201]^1,2^ |
| **Mitochondrial dysfunction** | *MGM1* [202]; *PIM1* [45]; CYT1 [59]; *MSW1* [44, 45]^*^; *MEF2* [203] | *sdhB* [204]; *dOpa1* [205]^3^ | *frh-1* [98, 99]^2,*^; *mev-1* [206, 207]; *gas-1* [208, 209]; *nduf-2.2* [208] | *Ucp2* [210]; *Risp* [211]^3,*^; *Bcs1l* [212]; *mSuv3* [213]^3^ |  |  |
| **DNA repair / genome integrity** | *DNA2* [214]; *SGS1* [215]; *SRS2* [216]; *RAD27* [45, 217]; *MSH6, CHL1, MRE11, MSH2* [45]; *RAD52, RAD50, RAD51, RAD57* [218] |  | *xpa-1* [219]^6^ | *Ercc1* [220]; *Ercc2* [221]; *Ku86 (=Ku80)* [222, 223]; *Ku70* [223]; *DNA-PKcs* [224]; *Top3beta* [225] |  | *RECQL4* [226]; *WRN* [227] |
| **Telomere integrity/length** | Long telomeres, *RIF1* [115]^^; *UTH4* [228] |  |  | Short telomeres [229] |  | Short telomeres [230]^1^ |
| **Other functions** | *SHM1, LYS9, NCR1,* *KGD1, APM1, SYM1, IDP2, GSY2, ALT2, SIZ1, PTC5, RNR3, ERG24, NUP170, SSO1, TWF1, SGT2, LHS1, ARF1, DHP5, SPE2, DBP1* [45]; *INP53* [32, 45]^*^; *RAS2* [231]; *ACH1* [232]; *RGS2* [126]; *SEL1, DIE2, KAR2* [119] | *Hk* [233]; *Mgat1* [234]; *Tpi* [235]; *dnr1* [236]; *Dcert* [237]; *Ald* [238] | *smn-1* [239]; *ptl-1* [240]; *mod-5* [132]; *jnk-1, jkk-1* [241]; *sgk-1* [17, 155, 242]; *sesn-1* [243]^6^; *aak-2* [244]; *nrf-4* [136]; *pnc-1* [245]^2^; *mnk-1* [47]^2^ | *eNos* [246]^*^; *Fn* [247]; *Ppm1d* [248]^*^; *Cdh1* [249]^1^; *Vimentin* [250]^1^ |  | *LMNA* [251, 252] |

^*^ both lifespan extension, shortening and no alteration of organismal lifespan have been described,^1^ studied in a cell culture model, ^2^ RNAi experiment, ^3^ animals heterozygous for the mutation were studied, ^4^ polymorphisms over-/underrepresented among long-lived populations, ^5^ over-expression of dominant-negative form, ^6^ RNAi and genetic mutant studied, ^7^ gain-of-function mutant

1. Lin, S.J., et al., *Calorie restriction extends Saccharomyces cerevisiae lifespan by increasing respiration.* Nature, 2002. **418**(6895): p. 344-8.

2. Klass, M.R., *Aging in the nematode Caenorhabditis elegans: major biological and environmental factors influencing life span.* Mech Ageing Dev, 1977. **6**(6): p. 413-29.

3. Liao, C.Y., et al., *Genetic variation in the murine lifespan response to dietary restriction: from life extension to life shortening.* Aging Cell, 2010. **9**(1): p. 92-5.

4. Rikke, B.A., et al., *Genetic dissection of dietary restriction in mice supports the metabolic efficiency model of life extension.* Exp Gerontol, 2010. **45**(9): p. 691-701.

5. Weindruch, R. and R.L. Walford, *Dietary restriction in mice beginning at 1 year of age: effect on life-span and spontaneous cancer incidence.* Science, 1982. **215**(4538): p. 1415-8.

6. McCay, C.M., M.F. Crowell, and L.A. Maynard, *The effect of retarded growth upon the length of life span and upon the ultimate body size. 1935.* Nutrition, 1989. **5**(3): p. 155-71; discussion 172.

7. Li, Y. and T.O. Tollefsbol, *p16(INK4a) suppression by glucose restriction contributes to human cellular lifespan extension through SIRT1-mediated epigenetic and genetic mechanisms.* PLoS One, 2011. **6**(2): p. e17421.

8. Lin, S.J., P.A. Defossez, and L. Guarente, *Requirement of NAD and SIR2 for life-span extension by calorie restriction in Saccharomyces cerevisiae.* Science, 2000. **289**(5487): p. 2126-8.

9. Tatar, M., et al., *A mutant Drosophila insulin receptor homolog that extends life-span and impairs neuroendocrine function.* Science, 2001. **292**(5514): p. 107-10.

10. Yamamoto, R. and M. Tatar, *Insulin receptor substrate chico acts with the transcription factor FOXO to extend Drosophila lifespan.* Aging Cell, 2011. **10**(4): p. 729-32.

11. Slack, C., et al., *Regulation of lifespan, metabolism, and stress responses by the Drosophila SH2B protein, Lnk.* PLoS Genet, 2010. **6**(3): p. e1000881.

12. Kenyon, C., et al., *A C. elegans mutant that lives twice as long as wild type.* Nature, 1993. **366**(6454): p. 461-4.

13. Klass, M.R., *A method for the isolation of longevity mutants in the nematode Caenorhabditis elegans and initial results.* Mech Ageing Dev, 1983. **22**(3-4): p. 279-86.

14. Rahman, M.M., et al., *Intracellular protein glycosylation modulates insulin mediated lifespan in C.elegans.* Aging (Albany NY), 2010. **2**(10): p. 678-90.

15. Mansisidor, A.R., et al., *A conserved PHD finger protein and endogenous RNAi modulate insulin signaling in Caenorhabditis elegans.* PLoS Genet, 2011. **7**(9): p. e1002299.

16. Hamilton, B., et al., *A systematic RNAi screen for longevity genes in C. elegans.* Genes Dev, 2005. **19**(13): p. 1544-55.

17. Alam, H., et al., *EAK-7 controls development and life span by regulating nuclear DAF-16/FoxO activity.* Cell Metab, 2010. **12**(1): p. 30-41.

18. Holzenberger, M., et al., *IGF-1 receptor regulates lifespan and resistance to oxidative stress in mice.* Nature, 2003. **421**(6919): p. 182-7.

19. Xu, J., et al., *Longevity effect of IGF-1R(+/-) mutation depends on genetic background-specific receptor activation.* Aging Cell, 2014. **13**(1): p. 19-28.

20. Conover, C.A. and L.K. Bale, *Loss of pregnancy-associated plasma protein A extends lifespan in mice.* Aging Cell, 2007. **6**(5): p. 727-9.

21. Selman, C., et al., *Evidence for lifespan extension and delayed age-related biomarkers in insulin receptor substrate 1 null mice.* FASEB J, 2008. **22**(3): p. 807-18.

22. Nojima, A., et al., *Haploinsufficiency of akt1 prolongs the lifespan of mice.* PLoS One, 2013. **8**(7): p. e69178.

23. Bonafe, M., et al., *Polymorphic variants of insulin-like growth factor I (IGF-I) receptor and phosphoinositide 3-kinase genes affect IGF-I plasma levels and human longevity: cues for an evolutionarily conserved mechanism of life span control.* J Clin Endocrinol Metab, 2003. **88**(7): p. 3299-304.

24. Simon, A.F., et al., *Steroid control of longevity in Drosophila melanogaster.* Science, 2003. **299**(5611): p. 1407-10.

25. Fisher, A.L. and G.J. Lithgow, *The nuclear hormone receptor DAF-12 has opposing effects on Caenorhabditis elegans lifespan and regulates genes repressed in multiple long-lived worms.* Aging Cell, 2006. **5**(2): p. 127-38.

26. Sun, L.Y., et al., *Growth hormone-releasing hormone disruption extends lifespan and regulates response to caloric restriction in mice.* Elife, 2013. **2**: p. e01098.

27. Flurkey, K., et al., *Lifespan extension and delayed immune and collagen aging in mutant mice with defects in growth hormone production.* Proc Natl Acad Sci U S A, 2001. **98**(12): p. 6736-41.

28. Coschigano, K.T., et al., *Deletion, but not antagonism, of the mouse growth hormone receptor results in severely decreased body weights, insulin, and insulin-like growth factor I levels and increased life span.* Endocrinology, 2003. **144**(9): p. 3799-810.

29. Kuramoto, K., et al., *Spontaneous dwarf rat: a novel model for aging research.* Geriatr Gerontol Int, 2010. **10**(1): p. 94-101.

30. Barbieri, M., et al., *Gender specific association of genetic variation in peroxisome proliferator-activated receptor (PPAR)gamma-2 with longevity.* Exp Gerontol, 2004. **39**(7): p. 1095-100.

31. Kaeberlein, M., et al., *Regulation of yeast replicative life span by TOR and Sch9 in response to nutrients.* Science, 2005. **310**(5751): p. 1193-6.

32. Smith, E.D., et al., *Quantitative evidence for conserved longevity pathways between divergent eukaryotic species.* Genome Res, 2008. **18**(4): p. 564-70.

33. Powers, R.W., 3rd, et al., *Extension of chronological life span in yeast by decreased TOR pathway signaling.* Genes Dev, 2006. **20**(2): p. 174-84.

34. Fabrizio, P., et al., *Regulation of longevity and stress resistance by Sch9 in yeast.* Science, 2001. **292**(5515): p. 288-90.

35. Kapahi, P., et al., *Regulation of lifespan in Drosophila by modulation of genes in the TOR signaling pathway.* Curr Biol, 2004. **14**(10): p. 885-90.

36. Hansen, M., et al., *Lifespan extension by conditions that inhibit translation in Caenorhabditis elegans.* Aging Cell, 2007. **6**(1): p. 95-110.

37. Pan, K.Z., et al., *Inhibition of mRNA translation extends lifespan in Caenorhabditis elegans.* Aging Cell, 2007. **6**(1): p. 111-9.

38. Vellai, T., et al., *Genetics: influence of TOR kinase on lifespan in C. elegans.* Nature, 2003. **426**(6967): p. 620.

39. Jia, K., D. Chen, and D.L. Riddle, *The TOR pathway interacts with the insulin signaling pathway to regulate C. elegans larval development, metabolism and life span.* Development, 2004. **131**(16): p. 3897-906.

40. Hansen, M., et al., *New genes tied to endocrine, metabolic, and dietary regulation of lifespan from a Caenorhabditis elegans genomic RNAi screen.* PLoS Genet, 2005. **1**(1): p. 119-28.

41. Harrison, D.E., et al., *Rapamycin fed late in life extends lifespan in genetically heterogeneous mice.* Nature, 2009. **460**(7253): p. 392-5.

42. Selman, C., et al., *Ribosomal protein S6 kinase 1 signaling regulates mammalian life span.* Science, 2009. **326**(5949): p. 140-4.

43. Steffen, K.K., et al., *Yeast life span extension by depletion of 60s ribosomal subunits is mediated by Gcn4.* Cell, 2008. **133**(2): p. 292-302.

44. Managbanag, J.R., et al., *Shortest-path network analysis is a useful approach toward identifying genetic determinants of longevity.* PLoS One, 2008. **3**(11): p. e3802.

45. Laschober, G.T., et al., *Identification of evolutionarily conserved genetic regulators of cellular aging.* Aging Cell, 2010. **9**(6): p. 1084-97.

46. Chiocchetti, A., et al., *Ribosomal proteins Rpl10 and Rps6 are potent regulators of yeast replicative life span.* Exp Gerontol, 2007. **42**(4): p. 275-86.

47. Syntichaki, P., K. Troulinaki, and N. Tavernarakis, *eIF4E function in somatic cells modulates ageing in Caenorhabditis elegans.* Nature, 2007. **445**(7130): p. 922-6.

48. Reis-Rodrigues, P., et al., *Proteomic analysis of age-dependent changes in protein solubility identifies genes that modulate lifespan.* Aging Cell, 2012. **11**(1): p. 120-7.

49. Tohyama, D., A. Yamaguchi, and T. Yamashita, *Inhibition of a eukaryotic initiation factor (eIF2Bdelta/F11A3.2) during adulthood extends lifespan in Caenorhabditis elegans.* FASEB J, 2008. **22**(12): p. 4327-37.

50. Stout, G.J., et al., *Insulin/IGF-1-mediated longevity is marked by reduced protein metabolism.* Mol Syst Biol, 2013. **9**: p. 679.

51. Chen, D., et al., *Longevity determined by developmental arrest genes in Caenorhabditis elegans.* Aging Cell, 2007. **6**(4): p. 525-33.

52. Kim, Y. and H. Sun, *Functional genomic approach to identify novel genes involved in the regulation of oxidative stress resistance and animal lifespan.* Aging Cell, 2007. **6**(4): p. 489-503.

53. Lakowski, B. and S. Hekimi, *Determination of life-span in Caenorhabditis elegans by four clock genes.* Science, 1996. **272**(5264): p. 1010-3.

54. Kruegel, U., et al., *Elevated proteasome capacity extends replicative lifespan in Saccharomyces cerevisiae.* PLoS Genet, 2011. **7**(9): p. e1002253.

55. Mehta, R., et al., *Proteasomal regulation of the hypoxic response modulates aging in C. elegans.* Science, 2009. **324**(5931): p. 1196-8.

56. Li, W., et al., *RLE-1, an E3 ubiquitin ligase, regulates C. elegans aging by catalyzing DAF-16 polyubiquitination.* Dev Cell, 2007. **12**(2): p. 235-46.

57. Dang, W., et al., *Histone H4 lysine 16 acetylation regulates cellular lifespan.* Nature, 2009. **459**(7248): p. 802-7.

58. Ryu, H.Y., B.H. Rhie, and S.H. Ahn, *Loss of the Set2 histone methyltransferase increases cellular lifespan in yeast cells.* Biochem Biophys Res Commun, 2014. **446**(1): p. 113-8.

59. Li, B., et al., *Identification of potential calorie restriction-mimicking yeast mutants with increased mitochondrial respiratory chain and nitric oxide levels.* J Aging Res, 2011. **2011**: p. 673185.

60. Larson, K., et al., *Heterochromatin formation promotes longevity and represses ribosomal RNA synthesis.* PLoS Genet, 2012. **8**(1): p. e1002473.

61. Greer, E.L., et al., *Members of the H3K4 trimethylation complex regulate lifespan in a germline-dependent manner in C. elegans.* Nature, 2010. **466**(7304): p. 383-7.

62. Ni, Z., et al., *Two SET domain containing genes link epigenetic changes and aging in Caenorhabditis elegans.* Aging Cell, 2012. **11**(2): p. 315-25.

63. Maures, T.J., et al., *The H3K27 demethylase UTX-1 regulates C. elegans lifespan in a germline-independent, insulin-dependent manner.* Aging Cell, 2011. **10**(6): p. 980-90.

64. Vora, M., et al., *Deletion of microRNA-80 activates dietary restriction to extend C. elegans healthspan and lifespan.* PLoS Genet, 2013. **9**(8): p. e1003737.

65. De Vaux, V., et al., *The Caenorhabditis elegans LET-418/Mi2 plays a conserved role in lifespan regulation.* Aging Cell, 2013. **12**(6): p. 1012-20.

66. Dellago, H., et al., *High levels of oncomiR-21 contribute to the senescence-induced growth arrest in normal human cells and its knock-down increases the replicative lifespan.* Aging Cell, 2013. **12**(3): p. 446-58.

67. Yoshida, R., et al., *Metabolomics-based systematic prediction of yeast lifespan and its application for semi-rational screening of ageing-related mutants.* Aging Cell, 2010. **9**(4): p. 616-25.

68. Alvers, A.L., et al., *Autophagy and amino acid homeostasis are required for chronological longevity in Saccharomyces cerevisiae.* Aging Cell, 2009. **8**(4): p. 353-69.

69. Kamei, Y., et al., *GABA metabolism pathway genes, UGA1 and GAD1, regulate replicative lifespan in Saccharomyces cerevisiae.* Biochem Biophys Res Commun, 2011. **407**(1): p. 185-90.

70. Boehm, M. and F. Slack, *A developmental timing microRNA and its target regulate life span in C. elegans.* Science, 2005. **310**(5756): p. 1954-7.

71. Thyagarajan, B., et al., *ETS-4 is a transcriptional regulator of life span in Caenorhabditis elegans.* PLoS Genet, 2010. **6**(9): p. e1001125.

72. Yang, P., et al., *A C-terminal truncated mutation of spr-3 gene extends lifespan in Caenorhabditis elegans.* Acta Biochim Biophys Sin (Shanghai), 2013. **45**(7): p. 540-8.

73. Chen, D., E.L. Thomas, and P. Kapahi, *HIF-1 modulates dietary restriction-mediated lifespan extension via IRE-1 in Caenorhabditis elegans.* PLoS Genet, 2009. **5**(5): p. e1000486.

74. Zhang, Y., et al., *The HIF-1 hypoxia-inducible factor modulates lifespan in C. elegans.* PLoS One, 2009. **4**(7): p. e6348.

75. Budovskaya, Y.V., et al., *An elt-3/elt-5/elt-6 GATA transcription circuit guides aging in C. elegans.* Cell, 2008. **134**(2): p. 291-303.

76. Mair, W., et al., *Lifespan extension induced by AMPK and calcineurin is mediated by CRTC-1 and CREB.* Nature, 2011. **470**(7334): p. 404-8.

77. Li, J., et al., *Caenorhabditis elegans HCF-1 functions in longevity maintenance as a DAF-16 regulator.* PLoS Biol, 2008. **6**(9): p. e233.

78. Brown-Borg, H.M., et al., *Dwarf mice and the ageing process.* Nature, 1996. **384**(6604): p. 33.

79. Bauer, J.H., et al., *Expression of dominant-negative Dmp53 in the adult fly brain inhibits insulin signaling.* Proc Natl Acad Sci U S A, 2007. **104**(33): p. 13355-60.

80. Olsen, A., M.C. Vantipalli, and G.J. Lithgow, *Checkpoint proteins control survival of the postmitotic cells in Caenorhabditis elegans.* Science, 2006. **312**(5778): p. 1381-5.

81. Carnero, A., et al., *p16INK4A and p19ARF act in overlapping pathways in cellular immortalization.* Nat Cell Biol, 2000. **2**(3): p. 148-55.

82. Kirley, S.D., et al., *Increased growth rate, delayed senescense and decreased serum dependence characterize cables-deficient cells.* Cancer Biol Ther, 2005. **4**(6): p. 654-8.

83. Bond, J.A., F.S. Wyllie, and D. Wynford-Thomas, *Escape from senescence in human diploid fibroblasts induced directly by mutant p53.* Oncogene, 1994. **9**(7): p. 1885-9.

84. Gollahon, L.S. and J.W. Shay, *Immortalization of human mammary epithelial cells transfected with mutant p53 (273his).* Oncogene, 1996. **12**(4): p. 715-25.

85. Bond, J., et al., *Evidence that transcriptional activation by p53 plays a direct role in the induction of cellular senescence.* Oncogene, 1996. **13**(10): p. 2097-104.

86. Wei, W., et al., *Loss of retinoblastoma but not p16 function allows bypass of replicative senescence in human fibroblasts.* EMBO Rep, 2003. **4**(11): p. 1061-6.

87. Noble, J.R., et al., *Association of extended in vitro proliferative potential with loss of p16INK4 expression.* Oncogene, 1996. **13**(6): p. 1259-68.

88. Fung, C., et al., *p16(INK) (4a) deficiency promotes DNA hyper-replication and genetic instability in melanocytes.* Pigment Cell Melanoma Res, 2013. **26**(2): p. 236-46.

89. Wheaton, K., et al., *BTG2 antagonizes Pin1 in response to mitogens and telomere disruption during replicative senescence.* Aging Cell, 2010. **9**(5): p. 747-60.

90. Gire, V., et al., *DNA damage checkpoint kinase Chk2 triggers replicative senescence.* EMBO J, 2004. **23**(13): p. 2554-63.

91. Mesquita, A., et al., *Caloric restriction or catalase inactivation extends yeast chronological lifespan by inducing H2O2 and superoxide dismutase activity.* Proc Natl Acad Sci U S A, 2010. **107**(34): p. 15123-8.

92. Van Raamsdonk, J.M. and S. Hekimi, *Deletion of the mitochondrial superoxide dismutase sod-2 extends lifespan in Caenorhabditis elegans.* PLoS Genet, 2009. **5**(2): p. e1000361.

93. Ayyadevara, S., et al., *Caenorhabditis elegans PI3K mutants reveal novel genes underlying exceptional stress resistance and lifespan.* Aging Cell, 2009. **8**(6): p. 706-25.

94. Migliaccio, E., et al., *The p66shc adaptor protein controls oxidative stress response and life span in mammals.* Nature, 1999. **402**(6759): p. 309-13.

95. Kharade, S.V., et al., *Mrg19 depletion increases S. cerevisiae lifespan by augmenting ROS defence.* FEBS Lett, 2005. **579**(30): p. 6809-13.

96. Heeren, G., et al., *The mitochondrial ribosomal protein of the large subunit, Afo1p, determines cellular longevity through mitochondrial back-signaling via TOR1.* Aging (Albany NY), 2009. **1**(7): p. 622-36.

97. Liu, J., et al., *Drosophila sbo regulates lifespan through its function in the synthesis of coenzyme Q in vivo.* J Genet Genomics, 2011. **38**(6): p. 225-34.

98. Vazquez-Manrique, R.P., et al., *Reduction of Caenorhabditis elegans frataxin increases sensitivity to oxidative stress, reduces lifespan, and causes lethality in a mitochondrial complex II mutant.* FASEB J, 2006. **20**(1): p. 172-4.

99. Ventura, N., et al., *Reduced expression of frataxin extends the lifespan of Caenorhabditis elegans.* Aging Cell, 2005. **4**(2): p. 109-12.

100. Wong, A., P. Boutis, and S. Hekimi, *Mutations in the clk-1 gene of Caenorhabditis elegans affect developmental and behavioral timing.* Genetics, 1995. **139**(3): p. 1247-59.

101. Yang, W. and S. Hekimi, *Two modes of mitochondrial dysfunction lead independently to lifespan extension in Caenorhabditis elegans.* Aging Cell, 2010. **9**(3): p. 433-47.

102. Feng, J., F. Bussiere, and S. Hekimi, *Mitochondrial electron transport is a key determinant of life span in Caenorhabditis elegans.* Dev Cell, 2001. **1**(5): p. 633-44.

103. Ren, Y., et al., *Reduction of mitoferrin results in abnormal development and extended lifespan in Caenorhabditis elegans.* PLoS One, 2012. **7**(1): p. e29666.

104. Lee, S.S., et al., *A systematic RNAi screen identifies a critical role for mitochondria in C. elegans longevity.* Nat Genet, 2003. **33**(1): p. 40-8.

105. Zuryn, S., et al., *Mitochondrial dysfunction in Caenorhabditis elegans causes metabolic restructuring, but this is not linked to longevity.* Mech Ageing Dev, 2010. **131**(9): p. 554-61.

106. Dillin, A., et al., *Rates of behavior and aging specified by mitochondrial function during development.* Science, 2002. **298**(5602): p. 2398-401.

107. Houtkooper, R.H., et al., *Mitonuclear protein imbalance as a conserved longevity mechanism.* Nature, 2013. **497**(7450): p. 451-7.

108. Tsang, W.Y., et al., *Mitochondrial respiratory chain deficiency in Caenorhabditis elegans results in developmental arrest and increased life span.* J Biol Chem, 2001. **276**(34): p. 32240-6.

109. Ventura, N. and S.L. Rea, *Caenorhabditis elegans mitochondrial mutants as an investigative tool to study human neurodegenerative diseases associated with mitochondrial dysfunction.* Biotechnol J, 2007. **2**(5): p. 584-95.

110. Maxwell, S., et al., *The SFT-1 and OXA-1 respiratory chain complex assembly factors influence lifespan by distinct mechanisms in C. elegans.* Longev Healthspan, 2013. **2**(1): p. 9.

111. Hoffmann, M., et al., *MICS-1 interacts with mitochondrial ATAD-3 and modulates lifespan in C. elegans.* Exp Gerontol, 2012. **47**(3): p. 270-5.

112. Liu, X., et al., *Evolutionary conservation of the clk-1-dependent mechanism of longevity: loss of mclk1 increases cellular fitness and lifespan in mice.* Genes Dev, 2005. **19**(20): p. 2424-34.

113. Dell'agnello, C., et al., *Increased longevity and refractoriness to Ca(2+)-dependent neurodegeneration in Surf1 knockout mice.* Hum Mol Genet, 2007. **16**(4): p. 431-44.

114. Rose, G., et al., *Two variants located in the upstream enhancer region of human UCP1 gene affect gene expression and are correlated with human longevity.* Exp Gerontol, 2011. **46**(11): p. 897-904.

115. Austriaco, N.R., Jr. and L.P. Guarente, *Changes of telomere length cause reciprocal changes in the lifespan of mother cells in Saccharomyces cerevisiae.* Proc Natl Acad Sci U S A, 1997. **94**(18): p. 9768-72.

116. Joeng, K.S., et al., *Long lifespan in worms with long telomeric DNA.* Nat Genet, 2004. **36**(6): p. 607-11.

117. Wright, W.E., et al., *Experimental elongation of telomeres extends the lifespan of immortal x normal cell hybrids.* EMBO J, 1996. **15**(7): p. 1734-41.

118. Fabrizio, P., et al., *Chronological aging-independent replicative life span regulation by Msn2/Msn4 and Sod2 in Saccharomyces cerevisiae.* FEBS Lett, 2004. **557**(1-3): p. 136-42.

119. Labunskyy, V.M., et al., *Lifespan extension conferred by endoplasmic reticulum secretory pathway deficiency requires induction of the unfolded protein response.* PLoS Genet, 2014. **10**(1): p. e1004019.

120. Suh, J.M., et al., *An RGS-containing sorting nexin controls Drosophila lifespan.* PLoS One, 2008. **3**(5): p. e2152.

121. Yang, Q., et al., *Role of Drosophila alkaline ceramidase (Dacer) in Drosophila development and longevity.* Cell Mol Life Sci, 2010. **67**(9): p. 1477-90.

122. Wang, D., et al., *Knockdown expression of eukaryotic initiation factor 5 C-terminal domain containing protein extends lifespan in Drosophila melanogaster.* Biochem Biophys Res Commun, 2014. **446**(2): p. 465-9.

123. Rogina, B., et al., *Extended life-span conferred by cotransporter gene mutations in Drosophila.* Science, 2000. **290**(5499): p. 2137-40.

124. Toivonen, J.M., et al., *No influence of Indy on lifespan in Drosophila after correction for genetic and cytoplasmic background effects.* PLoS Genet, 2007. **3**(6): p. e95.

125. Lin, Y.J., L. Seroude, and S. Benzer, *Extended life-span and stress resistance in the Drosophila mutant methuselah.* Science, 1998. **282**(5390): p. 943-6.

126. Lin, Y.R., et al., *Regulation of longevity by regulator of G-protein signaling protein, Loco.* Aging Cell, 2011. **10**(3): p. 438-47.

127. Nishimura, M., et al., *A dual role for integrin-linked kinase and beta1-integrin in modulating cardiac aging.* Aging Cell, 2014.

128. Feng, Y., et al., *FGT-1 is the major glucose transporter in C. elegans and is central to aging pathways.* Biochem J, 2013. **456**(2): p. 219-29.

129. Shen, L.L., et al., *Genes required for the functions of olfactory AWA neuron regulate the longevity of Caenorhabditis elegans in an insulin/IGF signaling-dependent fashion.* Neurosci Bull, 2010. **26**(2): p. 91-103.

130. Kumsta, C., et al., *Integrin-linked kinase modulates longevity and thermotolerance in C. elegans through neuronal control of HSF-1.* Aging Cell, 2013.

131. Oh, S.W., et al., *Identification of direct DAF-16 targets controlling longevity, metabolism and diapause by chromatin immunoprecipitation.* Nat Genet, 2006. **38**(2): p. 251-7.

132. Murakami, H. and S. Murakami, *Serotonin receptors antagonistically modulate Caenorhabditis elegans longevity.* Aging Cell, 2007. **6**(4): p. 483-8.

133. Huang, X., H. Zhang, and H. Zhang, *The zinc-finger protein SEA-2 regulates larval developmental timing and adult lifespan in C. elegans.* Development, 2011. **138**(10): p. 2059-68.

134. Ferguson, A.A., et al., *TATN-1 mutations reveal a novel role for tyrosine as a metabolic signal that influences developmental decisions and longevity in Caenorhabditis elegans.* PLoS Genet, 2013. **9**(12): p. e1004020.

135. Mouchiroud, L., et al., *Pyruvate imbalance mediates metabolic reprogramming and mimics lifespan extension by dietary restriction in Caenorhabditis elegans.* Aging Cell, 2011. **10**(1): p. 39-54.

136. Brejning, J., et al., *Loss of NDG-4 extends lifespan and stress resistance in Caenorhabditis elegans.* Aging Cell, 2014. **13**(1): p. 156-64.

137. Quach, T.K., et al., *Genome-wide microarrray analysis reveals roles for the REF-1 family member HLH-29 in ferritin synthesis and peroxide stress response.* PLoS One, 2013. **8**(3): p. e59719.

138. Palmitessa, A. and J.L. Benovic, *Arrestin and the multi-PDZ domain-containing protein MPZ-1 interact with phosphatase and tensin homolog (PTEN) and regulate Caenorhabditis elegans longevity.* J Biol Chem, 2010. **285**(20): p. 15187-200.

139. Lee, B.H. and K. Ashrafi, *A TRPV channel modulates C. elegans neurosecretion, larval starvation survival, and adult lifespan.* PLoS Genet, 2008. **4**(10): p. e1000213.

140. Yan, L., et al., *Type 5 adenylyl cyclase disruption increases longevity and protects against stress.* Cell, 2007. **130**(2): p. 247-58.

141. Enns, L.C., et al., *Disruption of protein kinase A in mice enhances healthy aging.* PLoS One, 2009. **4**(6): p. e5963.

142. Borras, C., et al., *RasGrf1 deficiency delays aging in mice.* Aging (Albany NY), 2011. **3**(3): p. 262-76.

143. Humbert, N., et al., *Regulation of ploidy and senescence by the AMPK-related kinase NUAK1.* EMBO J, 2010. **29**(2): p. 376-86.

144. Zhao, L., et al., *Common genetic variants of the beta2-adrenergic receptor affect its translational efficiency and are associated with human longevity.* Aging Cell, 2012. **11**(6): p. 1094-101.

145. Cui, H., et al., *Notch3 functions as a tumor suppressor by controlling cellular senescence.* Cancer Res, 2013. **73**(11): p. 3451-9.

146. Tresini, M., et al., *Modulation of replicative senescence of diploid human cells by nuclear ERK signaling.* J Biol Chem, 2007. **282**(6): p. 4136-51.

147. Mihaylova, V.T., et al., *The PTEN tumor suppressor homolog in Caenorhabditis elegans regulates longevity and dauer formation in an insulin receptor-like signaling pathway.* Proc Natl Acad Sci U S A, 1999. **96**(13): p. 7427-32.

148. Casellas, J. and J.F. Medrano, *Lack of Socs2 expression reduces lifespan in high-growth mice.* Age (Dordr), 2008. **30**(4): p. 245-9.

149. Pawlikowska, L., et al., *Association of common genetic variation in the insulin/IGF1 signaling pathway with human longevity.* Aging Cell, 2009. **8**(4): p. 460-72.

150. Willcox, B.J., et al., *FOXO3A genotype is strongly associated with human longevity.* Proc Natl Acad Sci U S A, 2008. **105**(37): p. 13987-92.

151. Donlon, T.A., et al., *FOXO3 gene variants and human aging: coding variants may not be key players.* J Gerontol A Biol Sci Med Sci, 2012. **67**(11): p. 1132-9.

152. Chateau, M.T., et al., *Klotho interferes with a novel FGF-signalling pathway and insulin/Igf-like signalling to improve longevity and stress resistance in Caenorhabditis elegans.* Aging (Albany NY), 2010. **2**(9): p. 567-81.

153. Kuro-o, M., et al., *Mutation of the mouse klotho gene leads to a syndrome resembling ageing.* Nature, 1997. **390**(6655): p. 45-51.

154. Razzaque, M.S., et al., *Premature aging-like phenotype in fibroblast growth factor 23 null mice is a vitamin D-mediated process.* FASEB J, 2006. **20**(6): p. 720-2.

155. Soukas, A.A., et al., *Rictor/TORC2 regulates fat metabolism, feeding, growth, and life span in Caenorhabditis elegans.* Genes Dev, 2009. **23**(4): p. 496-511.

156. Bayat, V., et al., *Mutations in the mitochondrial methionyl-tRNA synthetase cause a neurodegenerative phenotype in flies and a recessive ataxia (ARSAL) in humans.* PLoS Biol, 2012. **10**(3): p. e1001288.

157. Liu, H.Y. and C.M. Pfleger, *Mutation in E1, the ubiquitin activating enzyme, reduces Drosophila lifespan and results in motor impairment.* PLoS One, 2013. **8**(1): p. e32835.

158. Yun, C., et al., *Proteasomal adaptation to environmental stress links resistance to proteotoxicity with longevity in Caenorhabditis elegans.* Proc Natl Acad Sci U S A, 2008. **105**(19): p. 7094-9.

159. Marshall, H., et al., *Deficiency of the dual ubiquitin/SUMO ligase Topors results in genetic instability and an increased rate of malignancy in mice.* BMC Mol Biol, 2010. **11**: p. 31.

160. Mashimo, T., et al., *Progressive Purkinje cell degeneration in tambaleante mutant mice is a consequence of a missense mutation in HERC1 E3 ubiquitin ligase.* PLoS Genet, 2009. **5**(12): p. e1000784.

161. Cao, X., et al., *WW domain-containing E3 ubiquitin protein ligase 1 (WWP1) delays cellular senescence by promoting p27(Kip1) degradation in human diploid fibroblasts.* J Biol Chem, 2011. **286**(38): p. 33447-56.

162. Yates, K.E., et al., *Repression of the SUMO-specific protease Senp1 induces p53-dependent premature senescence in normal human fibroblasts.* Aging Cell, 2008. **7**(5): p. 609-21.

163. Juhasz, G., et al., *Atg7-dependent autophagy promotes neuronal health, stress tolerance, and longevity but is dispensable for metamorphosis in Drosophila.* Genes Dev, 2007. **21**(23): p. 3061-6.

164. Hars, E.S., et al., *Autophagy regulates ageing in C. elegans.* Autophagy, 2007. **3**(2): p. 93-5.

165. Hashimoto, Y., S. Ookuma, and E. Nishida, *Lifespan extension by suppression of autophagy genes in Caenorhabditis elegans.* Genes Cells, 2009. **14**(6): p. 717-26.

166. Melendez, A., et al., *Autophagy genes are essential for dauer development and life-span extension in C. elegans.* Science, 2003. **301**(5638): p. 1387-91.

167. Kang, H.T., et al., *Autophagy impairment induces premature senescence in primary human fibroblasts.* PLoS One, 2011. **6**(8): p. e23367.

168. Kaeberlein, M., M. McVey, and L. Guarente, *The SIR2/3/4 complex and SIR2 alone promote longevity in Saccharomyces cerevisiae by two different mechanisms.* Genes Dev, 1999. **13**(19): p. 2570-80.

169. Kennedy, B.K., et al., *Mutation in the silencing gene SIR4 can delay aging in S. cerevisiae.* Cell, 1995. **80**(3): p. 485-96.

170. Kanfi, Y., et al., *The sirtuin SIRT6 regulates lifespan in male mice.* Nature, 2012. **483**(7388): p. 218-21.

171. Mostoslavsky, R., et al., *Genomic instability and aging-like phenotype in the absence of mammalian SIRT6.* Cell, 2006. **124**(2): p. 315-29.

172. Ha, C.W., M.K. Sung, and W.K. Huh, *Nsi1 plays a significant role in the silencing of ribosomal DNA in Saccharomyces cerevisiae.* Nucleic Acids Res, 2012. **40**(11): p. 4892-903.

173. Walter, D., A. Matter, and B. Fahrenkrog, *Bre1p-mediated histone H2B ubiquitylation regulates apoptosis in Saccharomyces cerevisiae.* J Cell Sci, 2010. **123**(Pt 11): p. 1931-9.

174. Liu, N., et al., *The microRNA miR-34 modulates ageing and neurodegeneration in Drosophila.* Nature, 2012. **482**(7386): p. 519-23.

175. Xu, P., et al., *The Drosophila microRNA Mir-14 suppresses cell death and is required for normal fat metabolism.* Curr Biol, 2003. **13**(9): p. 790-5.

176. Gil, J., et al., *Polycomb CBX7 has a unifying role in cellular lifespan.* Nat Cell Biol, 2004. **6**(1): p. 67-72.

177. Holliday, R., *Strong effects of 5-azacytidine on the in vitro lifespan of human diploid fibroblasts.* Exp Cell Res, 1986. **166**(2): p. 543-52.

178. Fairweather, D.S., M. Fox, and G.P. Margison, *The in vitro lifespan of MRC-5 cells is shortened by 5-azacytidine-induced demethylation.* Exp Cell Res, 1987. **168**(1): p. 153-9.

179. Honda, S. and M. Matsuo, *5-Azacytidine shortens the in vitro lifespan of human diploid cells.* Cell Biol Int Rep, 1987. **11**(2): p. 141.

180. Duan, R., et al., *The RNA polymerase II Rpb4/7 subcomplex regulates cellular lifespan through an mRNA decay process.* Biochem Biophys Res Commun, 2013. **441**(1): p. 266-70.

181. Lazakovitch, E., et al., *nfi-I affects behavior and life-span in C. elegans but is not essential for DNA replication or survival.* BMC Dev Biol, 2005. **5**: p. 24.

182. An, J.H. and T.K. Blackwell, *SKN-1 links C. elegans mesendodermal specification to a conserved oxidative stress response.* Genes Dev, 2003. **17**(15): p. 1882-93.

183. Shen, L., et al., *Regulation of longevity by genes required for the functions of AIY interneuron in nematode Caenorhabditis elegans.* Mech Ageing Dev, 2010. **131**(11-12): p. 732-8.

184. Dubrovsky, Y.V., W.E. Samsa, and R.V. Kondratov, *Deficiency of circadian protein CLOCK reduces lifespan and increases age-related cataract development in mice.* Aging (Albany NY), 2010. **2**(12): p. 936-44.

185. Kondratov, R.V., et al., *Early aging and age-related pathologies in mice deficient in BMAL1, the core componentof the circadian clock.* Genes Dev, 2006. **20**(14): p. 1868-73.

186. Kapeta, S., N. Chondrogianni, and E.S. Gonos, *Nuclear erythroid factor 2-mediated proteasome activation delays senescence in human fibroblasts.* J Biol Chem, 2010. **285**(11): p. 8171-84.

187. Connell-Crowley, L., et al., *Drosophila lacking the Cdk5 activator, p35, display defective axon guidance, age-dependent behavioral deficits and reduced lifespan.* Mech Dev, 2007. **124**(5): p. 341-9.

188. Icreverzi, A., et al., *Drosophila cyclin D/Cdk4 regulates mitochondrial biogenesis and aging and sensitizes animals to hypoxic stress.* Cell Cycle, 2012. **11**(3): p. 554-68.

189. Baker, D.J., et al., *BubR1 insufficiency causes early onset of aging-associated phenotypes and infertility in mice.* Nat Genet, 2004. **36**(7): p. 744-9.

190. Barker, M.G., L.J. Brimage, and K.A. Smart, *Effect of Cu,Zn superoxide dismutase disruption mutation on replicative senescence in Saccharomyces cerevisiae.* FEMS Microbiol Lett, 1999. **177**(2): p. 199-204.

191. Unlu, E.S. and A. Koc, *Effects of deleting mitochondrial antioxidant genes on life span.* Ann N Y Acad Sci, 2007. **1100**: p. 505-9.

192. Van Zandycke, S.M., P.J. Sohier, and K.A. Smart, *The impact of catalase expression on the replicative lifespan of Saccharomyces cerevisiae.* Mech Ageing Dev, 2002. **123**(4): p. 365-73.

193. Phillips, J.P., et al., *Null mutation of copper/zinc superoxide dismutase in Drosophila confers hypersensitivity to paraquat and reduced longevity.* Proc Natl Acad Sci U S A, 1989. **86**(8): p. 2761-5.

194. Svensson, M.J. and J. Larsson, *Thioredoxin-2 affects lifespan and oxidative stress in Drosophila.* Hereditas, 2007. **144**(1): p. 25-32.

195. Tsuda, M., et al., *Loss of Trx-2 enhances oxidative stress-dependent phenotypes in Drosophila.* FEBS Lett, 2010. **584**(15): p. 3398-401.

196. Sanchez, D., et al., *Loss of glial lazarillo, a homolog of apolipoprotein D, reduces lifespan and stress resistance in Drosophila.* Curr Biol, 2006. **16**(7): p. 680-6.

197. Miranda-Vizuete, A., et al., *Lifespan decrease in a Caenorhabditis elegans mutant lacking TRX-1, a thioredoxin expressed in ASJ sensory neurons.* FEBS Lett, 2006. **580**(2): p. 484-90.

198. Morcos, M., et al., *Glyoxalase-1 prevents mitochondrial protein modification and enhances lifespan in Caenorhabditis elegans.* Aging Cell, 2008. **7**(2): p. 260-9.

199. Moskovitz, J., et al., *Methionine sulfoxide reductase (MsrA) is a regulator of antioxidant defense and lifespan in mammals.* Proc Natl Acad Sci U S A, 2001. **98**(23): p. 12920-5.

200. Li, Y., et al., *Dilated cardiomyopathy and neonatal lethality in mutant mice lacking manganese superoxide dismutase.* Nat Genet, 1995. **11**(4): p. 376-81.

201. Young, J.J., A. Patel, and P. Rai, *Suppression of thioredoxin-1 induces premature senescence in normal human fibroblasts.* Biochem Biophys Res Commun, 2010. **392**(3): p. 363-8.

202. Scheckhuber, C.Q., et al., *Unopposed mitochondrial fission leads to severe lifespan shortening.* Cell Cycle, 2011. **10**(18): p. 3105-10.

203. Callegari, S., et al., *The MEF2 gene is essential for yeast longevity, with a dual role in cell respiration and maintenance of mitochondrial membrane potential.* FEBS Lett, 2011. **585**(8): p. 1140-6.

204. Walker, D.W., et al., *Hypersensitivity to oxygen and shortened lifespan in a Drosophila mitochondrial complex II mutant.* Proc Natl Acad Sci U S A, 2006. **103**(44): p. 16382-7.

205. Tang, S., et al., *Heterozygous mutation of Opa1 in Drosophila shortens lifespan mediated through increased reactive oxygen species production.* PLoS One, 2009. **4**(2): p. e4492.

206. Ishii, N., et al., *A mutation in succinate dehydrogenase cytochrome b causes oxidative stress and ageing in nematodes.* Nature, 1998. **394**(6694): p. 694-7.

207. Ishii, N., et al., *A methyl viologen-sensitive mutant of the nematode Caenorhabditis elegans.* Mutat Res, 1990. **237**(3-4): p. 165-71.

208. Kayser, E.B., M.M. Sedensky, and P.G. Morgan, *The effects of complex I function and oxidative damage on lifespan and anesthetic sensitivity in Caenorhabditis elegans.* Mech Ageing Dev, 2004. **125**(6): p. 455-64.

209. Hartman, P.S., et al., *Mitochondrial mutations differentially affect aging, mutability and anesthetic sensitivity in Caenorhabditis elegans.* Mech Ageing Dev, 2001. **122**(11): p. 1187-201.

210. Andrews, Z.B. and T.L. Horvath, *Uncoupling protein-2 regulates lifespan in mice.* Am J Physiol Endocrinol Metab, 2009. **296**(4): p. E621-7.

211. Hughes, B.G. and S. Hekimi, *A mild impairment of mitochondrial electron transport has sex-specific effects on lifespan and aging in mice.* PLoS One, 2011. **6**(10): p. e26116.

212. Leveen, P., et al., *The GRACILE mutation introduced into Bcs1l causes postnatal complex III deficiency: a viable mouse model for mitochondrial hepatopathy.* Hepatology, 2011. **53**(2): p. 437-47.

213. Chen, P.L., et al., *Mitochondrial genome instability resulting from SUV3 haploinsufficiency leads to tumorigenesis and shortened lifespan.* Oncogene, 2013. **32**(9): p. 1193-201.

214. Hoopes, L.L., et al., *Mutations in DNA replication genes reduce yeast life span.* Mol Cell Biol, 2002. **22**(12): p. 4136-46.

215. Mankouri, H.W. and A. Morgan, *The DNA helicase activity of yeast Sgs1p is essential for normal lifespan but not for resistance to topoisomerase inhibitors.* Mech Ageing Dev, 2001. **122**(11): p. 1107-20.

216. Mankouri, H.W., T.J. Craig, and A. Morgan, *SGS1 is a multicopy suppressor of srs2: functional overlap between DNA helicases.* Nucleic Acids Res, 2002. **30**(5): p. 1103-13.

217. Ringvoll, J., et al., *Mutations in the RAD27 and SGS1 genes differentially affect the chronological and replicative lifespan of yeast cells growing on glucose and glycerol.* FEMS Yeast Res, 2007. **7**(6): p. 848-59.

218. Park, P.U., P.A. Defossez, and L. Guarente, *Effects of mutations in DNA repair genes on formation of ribosomal DNA circles and life span in Saccharomyces cerevisiae.* Mol Cell Biol, 1999. **19**(5): p. 3848-56.

219. Hyun, M., et al., *Longevity and resistance to stress correlate with DNA repair capacity in Caenorhabditis elegans.* Nucleic Acids Res, 2008. **36**(4): p. 1380-9.

220. Weeda, G., et al., *Disruption of mouse ERCC1 results in a novel repair syndrome with growth failure, nuclear abnormalities and senescence.* Curr Biol, 1997. **7**(6): p. 427-39.

221. Dolle, M.E., et al., *Increased genomic instability is not a prerequisite for shortened lifespan in DNA repair deficient mice.* Mutat Res, 2006. **596**(1-2): p. 22-35.

222. Vogel, H., et al., *Deletion of Ku86 causes early onset of senescence in mice.* Proc Natl Acad Sci U S A, 1999. **96**(19): p. 10770-5.

223. Li, H., et al., *Deletion of Ku70, Ku80, or both causes early aging without substantially increased cancer.* Mol Cell Biol, 2007. **27**(23): p. 8205-14.

224. Espejel, S., et al., *Shorter telomeres, accelerated ageing and increased lymphoma in DNA-PKcs-deficient mice.* EMBO Rep, 2004. **5**(5): p. 503-9.

225. Kwan, K.Y. and J.C. Wang, *Mice lacking DNA topoisomerase IIIbeta develop to maturity but show a reduced mean lifespan.* Proc Natl Acad Sci U S A, 2001. **98**(10): p. 5717-21.

226. Kitao, S., et al., *Mutations in RECQL4 cause a subset of cases of Rothmund-Thomson syndrome.* Nat Genet, 1999. **22**(1): p. 82-4.

227. Yu, C.E., et al., *Positional cloning of the Werner's syndrome gene.* Science, 1996. **272**(5259): p. 258-62.

228. Kennedy, B.K., et al., *Redistribution of silencing proteins from telomeres to the nucleolus is associated with extension of life span in S. cerevisiae.* Cell, 1997. **89**(3): p. 381-91.

229. Vera, E., et al., *The rate of increase of short telomeres predicts longevity in mammals.* Cell Rep, 2012. **2**(4): p. 732-7.

230. Counter, C.M., et al., *Telomere shortening associated with chromosome instability is arrested in immortal cells which express telomerase activity.* EMBO J, 1992. **11**(5): p. 1921-9.

231. Hlavata, L., et al., *Elevated Ras/protein kinase A activity in Saccharomyces cerevisiae reduces proliferation rate and lifespan by two different reactive oxygen species-dependent routes.* Aging Cell, 2008. **7**(2): p. 148-57.

232. Orlandi, I., N. Casatta, and M. Vai, *Lack of Ach1 CoA-Transferase Triggers Apoptosis and Decreases Chronological Lifespan in Yeast.* Front Oncol, 2012. **2**: p. 67.

233. Bushey, D., et al., *Sleep, aging, and lifespan in Drosophila.* BMC Neurosci, 2010. **11**: p. 56.

234. Sarkar, M., et al., *Null mutations in Drosophila N-acetylglucosaminyltransferase I produce defects in locomotion and a reduced life span.* J Biol Chem, 2006. **281**(18): p. 12776-85.

235. Gnerer, J.P., R.A. Kreber, and B. Ganetzky, *wasted away, a Drosophila mutation in triosephosphate isomerase, causes paralysis, neurodegeneration, and early death.* Proc Natl Acad Sci U S A, 2006. **103**(41): p. 14987-93.

236. Cao, Y., et al., *Dnr1 mutations cause neurodegeneration in Drosophila by activating the innate immune response in the brain.* Proc Natl Acad Sci U S A, 2013. **110**(19): p. E1752-60.

237. Rao, R.P., et al., *Ceramide transfer protein function is essential for normal oxidative stress response and lifespan.* Proc Natl Acad Sci U S A, 2007. **104**(27): p. 11364-9.

238. Miller, D., C. Hannon, and B. Ganetzky, *A mutation in Drosophila Aldolase causes temperature-sensitive paralysis, shortened lifespan, and neurodegeneration.* J Neurogenet, 2012. **26**(3-4): p. 317-27.

239. Briese, M., et al., *Deletion of smn-1, the Caenorhabditis elegans ortholog of the spinal muscular atrophy gene, results in locomotor dysfunction and reduced lifespan.* Hum Mol Genet, 2009. **18**(1): p. 97-104.

240. Chew, Y.L., et al., *PTL-1 regulates neuronal integrity and lifespan in C. elegans.* J Cell Sci, 2013. **126**(Pt 9): p. 2079-91.

241. Oh, S.W., et al., *JNK regulates lifespan in Caenorhabditis elegans by modulating nuclear translocation of forkhead transcription factor/DAF-16.* Proc Natl Acad Sci U S A, 2005. **102**(12): p. 4494-9.

242. Chen, A.T., et al., *Effects of Caenorhabditis elegans sgk-1 mutations on lifespan, stress resistance, and DAF-16/FoxO regulation.* Aging Cell, 2013. **12**(5): p. 932-40.

243. Yang, Y.L., et al., *SESN-1 is a positive regulator of lifespan in Caenorhabditis elegans.* Exp Gerontol, 2013. **48**(3): p. 371-9.

244. Apfeld, J., et al., *The AMP-activated protein kinase AAK-2 links energy levels and insulin-like signals to lifespan in C. elegans.* Genes Dev, 2004. **18**(24): p. 3004-9.

245. van der Horst, A., et al., *The Caenorhabditis elegans nicotinamidase PNC-1 enhances survival.* Mech Ageing Dev, 2007. **128**(4): p. 346-9.

246. Li, W., et al., *Premature death and age-related cardiac dysfunction in male eNOS-knockout mice.* J Mol Cell Cardiol, 2004. **37**(3): p. 671-80.

247. Muro, A.F., et al., *Regulated splicing of the fibronectin EDA exon is essential for proper skin wound healing and normal lifespan.* J Cell Biol, 2003. **162**(1): p. 149-60.

248. Nannenga, B., et al., *Augmented cancer resistance and DNA damage response phenotypes in PPM1D null mice.* Mol Carcinog, 2006. **45**(8): p. 594-604.

249. Li, M., et al., *The adaptor protein of the anaphase promoting complex Cdh1 is essential in maintaining replicative lifespan and in learning and memory.* Nat Cell Biol, 2008. **10**(9): p. 1083-9.

250. Tolstonog, G.V., et al., *Role of the intermediate filament protein vimentin in delaying senescence and in the spontaneous immortalization of mouse embryo fibroblasts.* DNA Cell Biol, 2001. **20**(9): p. 509-29.

251. De Sandre-Giovannoli, A., et al., *Lamin a truncation in Hutchinson-Gilford progeria.* Science, 2003. **300**(5628): p. 2055.

252. Eriksson, M., et al., *Recurrent de novo point mutations in lamin A cause Hutchinson-Gilford progeria syndrome.* Nature, 2003. **423**(6937): p. 293-8.
